# Anti-inflammatory effect of novel polypeptide WMX-8 through specific blockade of the IL17RA signaling pathway

**DOI:** 10.3389/fphar.2026.1842752

**Published:** 2026-07-24

**Authors:** Xinmin Wang, Hang Bao, Yuya Wang, Yalu Wang, Cheng Guo, Yanning Wu, Guotao Yang, Yongbin Xu, Yali Li

**Affiliations:** 1 School of Life Science and Technology, Inner Mongolia University of Science and Technology, Baotou, China; 2 Inner Mongolia Autonomous Region People’s Hospital, Hohhot, China; 3 The First Affiliated Hospital of Baotou Medical College, Inner Mongolia University of Science and Technology, Baotou, China

**Keywords:** autoimmune disease, IL17RA, inflammatory signaling pathway, peptide drug, targeted therapy

## Abstract

Interleukin-17 receptor A (IL17RA) acts as a core mediator of pro-inflammatory signaling and contributes crucially to the pathogenesis of autoimmune disorders including psoriasis and rheumatoid arthritis. Monoclonal antibodies against IL17RA have achieved clinical application, yet their clinical utility is restricted by multiple drawbacks: high production cost, complicated manufacturing procedures, mandatory injection delivery, and absent oral bioavailability. To overcome these bottlenecks, this study develops a novel inhibitory peptide WMX-8 intended to specifically disrupt the binding between IL17A and IL17RA. The polypeptide WMX-8 was rationally designed and chemically synthesized, followed by purification to obtain high-purity samples. A series of systematic in vitro biological assessments were conducted using IL17RA-expressing cell models (keratinocytes and monocyte-macrophages), alongside IL17RA-deficient cells as negative control groups. Biochemical and cellular assays verified that WMX-8 binds IL17RA with strong affinity and markedly suppresses the secretion of pro-inflammatory cytokines in IL17RA-positive keratinocytes and monocyte-macrophages. Its anti-inflammatory efficacy is equivalent to that of the reference anti-IL17RA monoclonal antibody. The inhibitory activity fully relies on IL17RA expression, since the anti-inflammatory effect disappears entirely in IL17RA-knockout cells. In addition, WMX-8 displays desirable pharmaceutical features, including low immunogenicity and convenient large-scale synthesis.This study validates the promising druggable properties of WMX-8 targeting the IL17A–IL17RA axis. Our findings lay a solid foundation for further preclinical investigation of peptide therapeutics against IL17A/IL17RA for the treatment of autoimmune diseases.

## 1 Introduction

The pathogenesis of autoimmune diseases often involves abnormal activation of the immune system and the persistence of chronic inflammation (Liu et al., 2025). Within this complex immune network, interleukin-17 (IL-17), particularly its prototypical member IL-17A, has been identified as a key cytokine driving pathological processes (Veldhoen, 2017). By promoting the release of pro-inflammatory mediators from various tissue cells, including keratinocytes, fibroblasts, and synovial cells, IL-17A plays a critical role in the formation and maintenance of lesions in diseases such as psoriasis, psoriatic arthritis, and ankylosing spondylitis (Zhao et al., 2023), (Liu L. et al., 2016). Its action depends on binding to a specific receptor complex on the cell surface. This receptor is a heterodimer, wherein the IL-17RA subunit is not only essential for IL-17A signal transduction (Salgueiro et al., 2024) but also serves as a central hub connecting immune responses and tissue inflammation due to its widespread expression in various tissues. Consequently, precise intervention in the IL-17A/IL-17RA signaling axis is regarded as an effective strategy for treating a range of autoimmune diseases (Wang et al., 2025).

Based on this scientific consensus, monoclonal antibody drugs targeting the IL-17 pathway, such as the IL-17A inhibitors Secukinumab and Ixekizumab, as well as the IL-17RA inhibitor Brodalumab, have achieved revolutionary success in the clinic. These biologics significantly neutralize pathological IL-17 signaling, leading to marked symptom improvement for many patients (Prest et al., 2023), (Saeki et al., 2022), (Papp et al., 2012). However, deeper clinical experience has revealed inherent limitations of these large molecule drugs. Firstly, the requirement for injectable administration causes inconvenience and imposes a long-term economic burden on patients, affecting treatment adherence (Liu S. et al., 2016), (Shaw et al., 2023). Secondly, although relatively infrequent, the risk of immunogenicity remains, where some patients may develop anti-drug antibodies, potentially leading to reduced efficacy over time (Du et al., 2017). More importantly, the significant proportion of non-responders or inadequate responders in the clinic suggests underlying complexities, such as IL-17A-independent inflammatory pathways or alternative receptor activation mechanisms. These unmet clinical needs urgently call for novel therapeutic modalities with new mechanisms of action, convenient administration, and the ability to overcome the shortcomings of existing therapies.

The current Frontier in drug development is expanding from traditional antibodies to diverse directions, including small molecules, peptides, and oligonucleotides (Luo and Xu, 2023), (Yen and Lin, 2025), (Reich and Menter, 2018). Among these, therapeutic peptides have garnered significant attention due to their unique advantages (Zhu et al., 2024). Peptide molecules bridge the gap between small molecule chemicals and monoclonal antibodies (Zhang et al., 2016). Compared to small molecules, peptides generally bind to targets with higher affinity and specificity, making them particularly suitable for inhibiting challenging targets like protein-protein interactions (PPIs) (Zhang and Wang, 2022), (Sun and Davis, 2021). Compared to antibodies, peptides have a smaller molecular weight, potentially better tissue penetration, typically lower immunogenicity risk, and can be produced on a large scale through chemical synthesis, offering significant cost-effectiveness (Liu and Zhang, 2023). Therefore, developing functional peptides that precisely block the IL-17A/IL-17RA interaction interface holds promise not only as a more patient-friendly potential treatment but also as a novel tool for gaining deeper insights into the fine regulatory mechanisms of this critical signaling pathway (Ting et al., 2018).

However, applying peptide drugs clinically, especially for blocking high-affinity interactions like IL-17A/IL-17RA, faces several key scientific challenges (Fotiadou and Papp, 2022). The primary challenge is balancing activity and stability. Natural linear peptides are susceptible to rapid degradation by proteases in the body, resulting in a very short half-life. A core aspect of successful peptide drug development is how to use rational drug design strategies (e.g., cyclization, incorporation of non-natural amino acids, PEGylation) to significantly enhance metabolic stability and plasma half-life while maintaining high biological activity. Secondly, there is the issue of delivery efficiency. Although peptide tissue penetration may be improved compared to antibodies, ensuring effective delivery to disease target tissues like skin and joints, and efficient entry into the cellular microenvironment to competitively inhibit IL-17A, still requires in-depth exploration. Furthermore, systematic evaluation of the precise molecular mechanism, binding kinetics, and functional efficacy of the peptide molecule binding to its target, IL-17RA, is a crucial bridge connecting initial discovery to subsequent development.

Addressing the above background and challenges, this study aims to design and develop a novel peptide inhibitor capable of efficiently targeting IL-17RA and blocking the IL-17A signaling pathway.

Based on previously reported high-resolution crystal structure data of the IL-17A/IL-17RA complex, which identified key interfacial residues (e.g., Arg^49^, Gln^85^ in IL-17A and Lys^155^, Glu^186^ in IL-17RA) that are critical for ligand-receptor binding and downstream signaling activation (Fotiadou and Papp, 2022; Liu et al., 2013).

Utilizing a targeted peptide design strategy mimicking the binding epitope of IL-17A, we designed and synthesized a candidate peptide molecule named WMX-8. This research integrates biophysical techniques (such as ELISA) with functional analyses at the cellular level to systematically evaluate key characteristics of WMX-8: (1) its binding affinity and specificity for the IL17RA protein; (2) its potential regulatory effect on IL-17A-associated downstream signaling pathways including NF-κB; and (3) its capacity to suppress the production of key pro-inflammatory cytokines and chemokines (e.g., IL-6, CXCL1/IL-8, MMP-9).

This work is intended to establish a new experimental peptide candidate for preclinical investigation of autoimmune diseases, and further verify the feasibility of the IL17RA-targeted peptide blocking strategy. It lays the foundation for subsequent optimization of more stable and efficient peptide variants. Ultimately, we hope such research will provide new ideas for optimizing existing antibody-based intervention strategies and developing novel targeted molecules.

## 2 Methods

### 2.1 Ethics approval

All animal experiments were performed in accordance with the Guide for the Care and Use of Laboratory Animals published by the US National Institutes of Health (NIH Publication No. 85–23, revised 2011). The experimental protocols were approved by the Ethics Committee for Animal Experimentation of Inner Mongolia University of Science and Technology (Approval No [SCXK 2024-0001]).

### 2.2 Synthesis and detection of WMX-8

Peptides were synthesized by solid-phase synthesis using the Focus XC system (AAPPTec, USA). In the coupling reaction, 1-hydroxybenzotriazole (HOBt) and N,N′-diisopropylcarbodiimide (DIC) were used as coupling agents, and piperidine (PIP) was used as a deprotecting agent. After synthesis, the peptides were cleaved with 90% trifluoroacetic acid (TFA), and the crude product was precipitated with ether. The crude peptides were purified by high-performance liquid chromatography (HPLC), and their purity and molecular weight were verified by HPLC and electrospray ionization mass spectrometry (ESI-MS), respectively. Additionally, fluorescein isothiocyanate (FITC) was conjugated to WMX-8 through the Acp active group to prepare FITC-WMX-8 peptides, and the products were purified and analyzed through the same process.

### 2.3 Cell culture

The HaCaT human immortalized keratinocyte cell line and RAW264.7 murine mononuclear macrophage cell line were obtained from the Cell Bank of the Chinese Academy of Sciences (Shanghai, China). HaCaT and RAW264.7 cells were cultured in RPMI 1640 medium (Gibco, USA), which was supplemented with 100 μg/mL streptomycin, 50 μg/mL penicillin, 2,200 μg/mL sodium bicarbonate (NaHCO_3_) and 10% (v/v) fetal bovine serum (FBS) (Gibco, USA). All cells were maintained in a humidified incubator at 37 °C with 5% CO_2_.

### 2.4 Generation of IL17RA-deficient cells

IL17RA-deficient cells were generated using small interfering RNA (siRNA) transfection, with cells adjusted to a density of 1 × 10^6^ cells/mL; 1 day prior to transfection, cells were seeded into 6-well plates at 40% confluency, and the culture medium was replaced with 2 mL of RPMI 1640 medium (Gibco, USA) 4 h before transfection. For each well, 5 μL of 20 μM siRNA solution was first mixed with 35 μL of CALNP RNAi *in vitro* Reagent A, followed by the addition of 10 μL of CALNP RNAi *in vitro* Reagent B, and the mixture was allowed to stand for 5 min before 150 μL of cell culture medium was added to bring the total volume to 200 μL (designated as Solution A), which was then added to the 6-well plates containing 1.8 mL of culture medium to achieve a final siRNA concentration of 50 nM, with all siRNAs, including the control siRNA, purchased from HyCyte (China). After incubating the transfection mixture at room temperature for 20 min, it was applied to the cells, the medium was replaced with fresh RPMI 1640 medium 6 h post-transfection, and cells were harvested for analysis 48 h after transfection, with the IL17RA siRNA sequences being as follows: sense strand 5′-GGC​UGA​GCU​GCA​GAG​UCA​ATT-3′ and antisense strand 5′-UUG​ACU​CUG​CAG​CUC​AGC​CTT-3’.

### 2.5 Real-time qPCR

For real-time quantitative polymerase chain reaction (qPCR), total RNA was extracted using Trizol reagent (Invitrogen, USA) and then reverse-transcribed into complementary DNA (cDNA) with the PrimeScript RT Kit (Invitrogen, USA) in accordance with the manufacturer’s instructions; subsequently, the mRNA expression levels of the target genes were quantified using SYBR Green Master Mix (Takara Bio Inc., Japan) on the QuantStudio Flex Real-Time PCR System (Life Technologies, Singapore).

### 2.6 Competitive enzyme-linked immunosorbent assay (ELISA)

For competitive enzyme-linked immunosorbent assay (ELISA), recombinant human IL-17RA protein (R&D Systems, USA) was coated onto 96-well microplates at 4 °C overnight and then blocked with 1% bovine serum albumin (BSA, Sigma-Aldrich, USA) at room temperature for 1 h in accordance with standard protocols; subsequently, serially diluted concentrations of WMX-8, together with a fixed concentration of biotin-labeled IL-17A (R&D Systems, USA), were added to each well, with a scrambled-sequence negative control peptide and excess unlabeled IL-17A positive control included to assess non-specific binding and competitive inhibition. After incubation at 37 °C for 2 h, the plates were washed, and horseradish peroxidase (HRP)-conjugated streptavidin (Thermo Fisher Scientific, USA) was added, followed by color development with 3,3′,5,5′-tetramethylbenzidine (TMB) substrate (Sigma-Aldrich, USA); the absorbance at 450 nm was measured using a Multiskan FC Microplate Photometer (Thermo Fisher Scientific, USA), and the half-maximal inhibitory concentration (EC50) of WMX-8 was calculated by fitting the competitive binding curve using GraphPad Prism 9 software (GraphPad Software, USA).

### 2.7 Flow cytometry analysis

Flow cytometry was used to evaluate the binding of peptide WMX-8 to cells; briefly, cells were digested from culture flasks with 0.25% trypsin (Seville Bio, China), then the cell suspension was counted using a hemocytometer (LOGOS BIOSYSTEMS, South Korea), the cell suspension corresponding to 1 × 10^6^ cells was taken and centrifuged again to remove the supernatant, 3 mL of BSA was added to the 1 × 10^6^ cells for incubation, followed by resuspension in PBS, then fluorescent WMX-8 peptide and fluorescent antibody (Proteintech, China) were added for incubation, and after resuspension in PBS, flow cytometry detection was performed using a flow cytometer (BD, USA).

### 2.8 Immunofluorescence analysis

IL17RA-expressing and IL17RA-deficient cells were separately seeded into 6-well plates and cultured to 40% confluency, then fixed with 4% paraformaldehyde and washed with PBS, followed by the addition of Hoechst, fluorescent peptide and fluorescent antibody (Proteintech, China) for co-incubation for 30 min; after incubation, the cells were washed three times with PBS, 1 mL of PBS was added subsequently, and the cells were directly observed and photographed under a fluorescence microscope (Nikon, Japan).

### 2.9 Cytotoxicity assay

IL17RA-expressing and IL17RA-deficient cells (1 × 10^4^ cells/mL) were separately seeded into 96-well plates, after which WMX-8 at various concentrations was added to the culture medium with a volume of 100 μL per well; 24 h later, 10 μL of Cell Counting Kit-8 (CCK-8) reagent (Beyotime, Shanghai, China) was added to each well and incubated for 4 h, and the absorbance was measured at 450 nm using a Multiskan Microplate Reader (Thermo Fisher Scientific, USA).

### 2.10 Cell motility assay

The wild-type and IL17RA-knockdown types of the two cell lines were separately seeded into 6-well plates and cultured for 24 h to reach a cell confluency of approximately 70%. This assay was designed to investigate the effect of WMX-8 on the baseline migratory behavior of cells, rather than on IL-17A-induced migration. Then, scratches were made on the surface of the wells with a pipette tip, with an average of three scratches per well, followed by gentle washing twice with PBS to remove detached cells. Subsequently, serum-free medium containing WMX-8 peptide (without exogenous IL-17A stimulation) was added to the wells, while serum-free medium alone was used as a control. The cell migration status was recorded at 0, 24, and 48 h after scratching (Luo and Xu, 2023).

### 2.11 *In vitro* anti-inflammatory assay

IL17RA-expressing and IL17RA-deficient cells, including HaCaT and RAW264.7 cells, were seeded into 6-well plates at a density of 1 × 10^5^ cells per well; after 24 h of culture, the cells were incubated with 100 nM WMX-8 peptide or 100 nM anti-IL17RA functional antibody (R&D Systems,MAB4481, USA) at 37 °C for 30 min, and subsequently, the aforementioned cells were co-cultured with 100 nM recombinant IL17A protein (Sigma-Aldrich, H7791, USA) at 37 °C for 24 h. It has been well documented that human IL-17A exhibits reliable cross-reactivity with murine IL17RA and can effectively trigger inflammatory responses in murine macrophages (Chen et al., 2013). At 6, 12, 18 and 24 h post the initiation of co-culture, the concentrations of corresponding cytokines in the cell supernatants were determined separately: IL-6, IL-8 and MMP-9 were detected in HaCaT cell supernatants, while tumor necrosis factor-α (TNF-α), MMP-9, IL-6 and CXCL1 were measured in RAW264.7 cell supernatants. All the above detections were performed using the corresponding enzyme-linked immunosorbent assay (ELISA) kits (Beyotime, Shanghai, China), and the experimental procedures were carried out in accordance with the manufacturer’s instructions.

### 2.12 CIA model establishment and in vivo drug administration

Specific pathogen-free female SD rats (6–8 weeks old, weighing 180–220 g) were selected for this study. The collagen-induced arthritis (CIA) model was established by subcutaneous injection at the base of the tail with an emulsion of equal volumes of bovine type II collagen (2 mg/mL, dissolved in 0.05 M acetic acid) and complete Freund’s adjuvant, at a dose of 0.1 mL per rat (200 μg collagen per rat). A booster immunization with the same emulsion (0.1 mL/rat) was administered via tail vein injection on day 7 after the primary immunization. The joint swelling and motor activity of rats were observed daily, and a rat was considered to be successfully modeled when its arthritis index (AI) reached no less than 4.

Rats with successful modeling were randomly divided into three groups (5 rats per group): model control group, WMX-8 peptide treatment group and methotrexate (MTX) positive control group. Another 5 unmodeled rats were assigned to the normal control group. The whole experimental period lasted for 28 days, and sample collection was scheduled on day 0, 7, 14, 21 and 28 after modeling.

Drug administration started on the day after model establishment. WMX-8 peptide and anti-IL17RA functional antibody (R&D Systems, Cat# MAB4481, USA) were dissolved in sterile normal saline and delivered via intraplantar injection (i.pl.) into the inflamed hind paw. WMX-8 peptide and methotrexate (MTX) were dissolved in sterile normal saline and delivered via intraplantar injection (i.pl.) into the inflamed hind paw. WMX-8 peptide was given at a dose of 100 nmol/rat with an injection volume of 50 μL per rat; MTX was administered with the same injection volume. Both treatments were performed three times a week for 28 consecutive days. The normal control group and model control group received an equal volume of sterile normal saline via the same route.

### 2.13 *In vivo* anti-inflammatory assay

For sample collection, rats were anesthetized by intraperitoneal injection of 10% (w/v) chloral hydrate at a dose of 0.3 mL per 100 g body weight. Blood was collected from the abdominal aorta and centrifuged to separate serum. Joint cavity fluid was collected via aseptic puncture. Subsequently, the rats were sacrificed, and the synovial tissue of lesioned knee joints was dissected. The tissue was homogenized in pre-cooled sterile normal saline at a ratio of 1:10 (w/v), followed by centrifugation at 12,000 r/min for 15 min at 4 °C. The supernatant was collected for subsequent detection. The concentrations of IL-6, MMP-9 and CXCL1 in serum, joint cavity fluid and synovial tissue homogenate supernatant were measured using corresponding enzyme-linked immunosorbent assay (ELISA) kits (Beyotime, Shanghai, China), and all assays were performed in strict accordance with the manufacturer’s instructions.

### 2.14 Open field test for detecting spontaneous locomotor activity of rats

The experiment was performed in an open field test arena with dimensions of 50 cm × 50 cm × 30 cm, the bottom of which was divided into 25 equal-area grids; the arena was equipped with a video tracking system for animal behavior, and fresh middle segments of *Tenebrio molitor* (1 mm in length) were pre-prepared and placed in the middle area of partial grids (one segment per grid). The animals used here were those that had been grouped and modeled in the above experiments. Rats from the normal control group, CIA model control group, CIA + methotrexate (MTX) group and CIA + WMX-8 peptide group (5 rats per group)were sequentially placed at the center of the arena, and the video tracking system was activated to record their behaviors within 5 min. After the test of each rat, the arena was thoroughly cleaned with 75% ethanol to eliminate olfactory cues. Each rat was independently subjected to the test 5 times, and the average values of the behavioral indices were calculated for subsequent analysis. Two behavioral indices were recorded and analyzed by the tracking system: the number of grid crossings (defined as the times of all four limbs completely crossing the grid boundary) and the number of consumed *T. molitor* middle segments (defined as the quantity of segments successfully ingested by rats).

### 2.15 Rat limb grip strength test

The test was performed using a grip strength meter equipped with a metal grid bar (accuracy: 0.01 g) on Rats from the normal control group, CIA model control group, CIA + methotrexate (MTX) group and CIA + WMX-8 peptide group (5 rats per group) For the test, the caudal end of each rat was gently grasped to guide its forelimbs to grip the metal grid bar, then the rat was pulled slowly backward, and the maximum pulling force at the moment the rat released the grid bar was recorded. Each rat was tested independently for 5 times; abnormal peak values caused by struggling were excluded, and the average value was calculated, which was used to reflect the grip strength level of the rats.

### 2.16 Joint range of motion (ROM) test

Joint Range of Motion (ROM) test was performed to evaluate the joint flexibility of rats, and Rats from the normal control group, CIA model control group, CIA + methotrexate (MTX) group and CIA + WMX-8 peptide group (5 rats per group) were selected for the test, with small protractors and adhesive tape used as experimental instruments. Firstly, the rats’ bodies were fixed with adhesive tape, and their ankle joints and knee joints were tested separately; during the operation, the corresponding limbs were gently held, and the joints were slowly extended and flexed to the maximum angles without the rats showing struggling responses, the extension angles and flexion angles of the joints were recorded with the protractors, and the ROM of the corresponding joints was calculated according to the formula: ROM = flexion angle − extension angle. The ankle and knee joints of each rat were tested repeatedly 5 times respectively, and the average values of the 5 measurements were taken as the final ROM data of the corresponding joints.

### 2.17 Body weight change monitoring in rats

Body weight changes of SD rats in each group were monitored synchronously throughout the experiment; SD rats were assigned to the normal control group, CIA model control group, CIA + methotrexate (MTX) group and CIA + WMX-8 peptide group. Body weight was measured every 7 days starting from the modeling day (day 0), with the detection time points at day 0, 7, 14, 21 and 28. Each measurement was conducted at a fixed time window of 8:00–9:00 a.m.; all rats were fasted for 1 h prior to weighing, and the body weight of each rat was measured using an electronic balance with an accuracy of 0.1 g, with all raw weight data recorded directly.

### 2.18 Joint swelling measurement (circumference method)

This experiment was carried out using female SD rats with established CIA models, which had been divided into four groups in the preceding study: normal control group, CIA model group, CIA + methotrexate (MTX) group and CIA + WMX-8 peptide groupBoth MTX and WMX-8 peptide were prepared with sterile normal saline and delivered via intraplantar injection (i.pl.) into the inflamed hind paw at an injection volume of 50 μL per rat. Treatments were initiated on the day after model establishment and conducted three times per week for a total of 28 days. Joint circumference measurements were performed every 7 days on day 0, 7, 14, 21 and 28. A flexible inelastic tape measure (accuracy: 0.1 mm) was used to measure the circumference at the maximal swelling site of the ankle joint and the midpoint of the knee joint. Each joint was measured five times, and the average value was recorded. Extraneous compression on the joints was strictly prohibited during measurement. All measurements were completed by a single operator to ensure consistent operation.

### 2.19 Arthritis scoring (0–16 points)

Arthritis scoring was performed synchronously with joint swelling measurement, once every 7 days starting from the modeling day (day 0) at the time points of day 0, 7, 14, 21 and 28, adopting a 0–16 point scoring system (the total score was the sum of the scores of the four paws after scoring each paw individually). The specific scoring criteria were as follows: 0 points (no erythema or swelling); 1 point (mild erythema or swelling in a single digit); 2 points (erythema and swelling in multiple digits); 3 points (erythema and swelling in the ankle/wrist joint); 4 points (erythema and swelling in the entire paw (digits + ankle/wrist) with impaired joint flexion). The four paws of each rat were visually observed by the same experimenter, each paw was scored independently according to the above criteria, and the scores were summed up, which was defined as the arthritis score of the rat.

### 2.20 HE staining experimental method

On day 28, after all preceding experimental measurements were finished, rats from each group were euthanized under general anesthesia induced by intraperitoneal injection of 10% (w/v) chloral hydrate (0.3 mL per 100 g body weight). Ankle synovial tissues were aseptically isolated and surrounding connective tissues were removed. The tissues were fixed in 4% paraformaldehyde for 24–48 h, followed by gradient ethanol dehydration, xylene clearing, and paraffin embedding. Serial sections (4–5 μm thick) were cut and baked at 60 °C for 2 h to enhance adhesion to slides. After deparaffinization in xylene and rehydration in gradient ethanol, sections were stained with hematoxylin-eosin (HE): nuclei were stained with hematoxylin for 5–8 min, differentiated in hydrochloric acid alcohol for 30 s, and blued in running water for 5 min; cytoplasm was then stained with eosin for 2–3 min. Finally, sections were dehydrated, cleared, and mounted with neutral gum, and the pathological morphology of synovial tissues was observed and recorded under a light microscope. Subsequently, the sections were scored using a 0–4 scale based on the overall degree of pathological damage in the synovial tissue. A score of 0 indicates intact synovial structure without hyperplasia, inflammatory infiltration, pannus formation, or cartilage/bone destruction. A score of 1 indicates mild synovial hyperplasia with a small amount of inflammatory cell infiltration, and no obvious pannus formation or cartilage destruction. A score of 2 indicates moderate synovial hyperplasia with a moderate amount of inflammatory cell infiltration, with a small amount of pannus visible and no obvious cartilage destruction. A score of 3 indicates severe synovial hyperplasia with a large amount of inflammatory cell infiltration, prominent pannus formation, and mild cartilage/bone destruction. A score of 4 indicates extremely severe synovial hyperplasia with a large amount of inflammatory cell infiltration, extensive pannus formation, and severe cartilage/bone destruction.

### 2.21 Immunohistochemistry (IHC) staining

For immunohistochemical (IHC) staining, formalin-fixed paraffin-embedded synovial tissue sections were deparaffinized in xylene and rehydrated through a graded ethanol series. Antigen retrieval was performed via high-pressure heating in citrate buffer (pH 6.0) for 15 min. Endogenous peroxidase activity was quenched with 3% hydrogen peroxide at room temperature for 10 min, followed by blocking with 5% bovine serum albumin (BSA) for 30 min to minimize non-specific binding. Sections were incubated with primary antibodies against CXCL2, MMP-1, and NF-κB-p65 at 4 °C overnight, then with horseradish peroxidase (HRP)-conjugated secondary antibodies at room temperature for 30 min. Immunoreactivity was visualized using 3,3′-diaminobenzidine (DAB) chromogen, and sections were counterstained with hematoxylin, dehydrated, cleared, and mounted. Protein localization and staining intensity were evaluated under a light microscope. Subsequently, the sections were scored using a 0–4 scale, with comprehensive evaluation based on the overall staining intensity and positive extent of pro-inflammatory factors (CXCL2, MMP-1, and NF-κB-p65). A score of 0 indicates no obvious positive staining, or only a very small amount of scattered staining. A score of 1 indicates a small amount of scattered positive staining with weak intensity. A score of 2 indicates a moderate amount of positive staining with moderate intensity. A score of 3 indicates a large amount of positive staining with strong intensity. A score of 4 indicates extensive strong positive staining that covers almost the entire tissue area.

### 2.22 Statistical analysis

Statistical analysis was performed using GraphPad Prism 8.0 (GraphPad Software, CA). All data are expressed as the mean ± standard error of the mean (SEM) from at least three independent experiments, with biological replicates (n = 2 or 3) per experiment. For comparisons among three or more groups under a single factor, one-way analysis of variance (ANOVA) was performed, followed by Bonferroni *post hoc* multiple comparisons to control the family-wise type I error rate. For experiments with two independent factors (e.g., cell type and treatment), two-way ANOVA was applied to evaluate both main effects and interaction effects, followed by Bonferroni *post hoc* tests. Differences were considered statistically significant at *P < 0.05, **P < 0.01, ***P < 0.001.

## 3 Results

### 3.1 1Structure and synthesis of peptides of WMX-8

**FIGURE 1:**
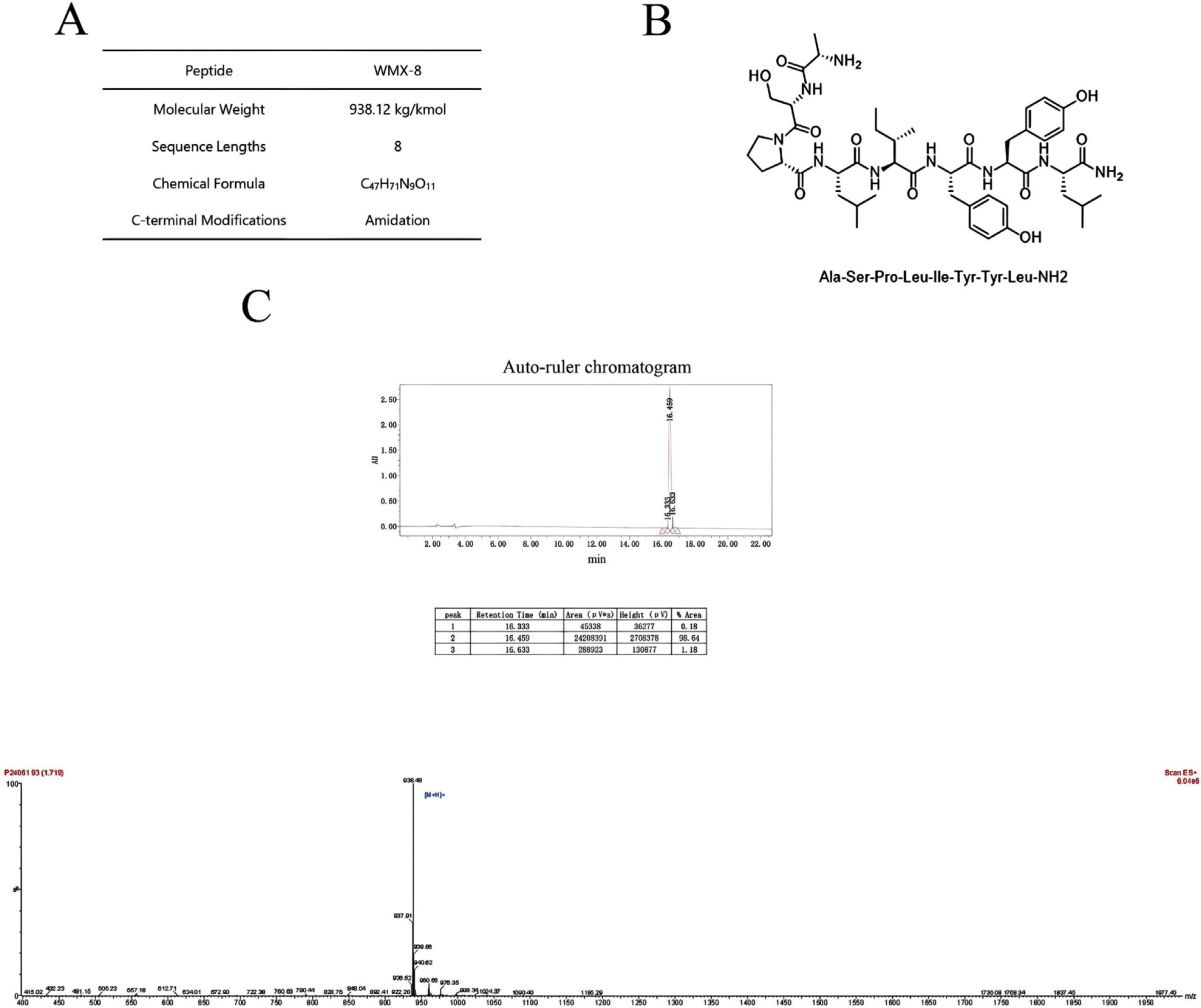
Structure and synthesis of peptides **(A)** Physicochemical properties of the peptides. **(B)** Chemical structure of peptide WMX-8. **(C)** Analysis of peptide WMX-8 by high-performance liquid chromatography (HPLC, up) and electrospray ionization mass spectrometry (ESI-MS, down).

Accumulating studies have established that aberrant activation of the IL17A-IL17RA signaling pathway is primarily driven and precisely regulated by the IL17A cytokine; accordingly, the present study was rationally designed targeting the IL17A-IL17RA binding interface to develop a peptide ligand, denoted as WMX-8, which possesses the amino acid sequence ASPLIYYL-NH_2_ (chemical structure depicted in Figure 1B), was synthesized employing solid-phase peptide synthesis (SPPS) via the Focus XC system, with its chromatographic purity quantified via high-performance liquid chromatography (HPLC) and molecular weight validated using electrospray ionization mass spectrometry (ESI-MS) (as presented in Figures 1C,D), while Figure 1A verifies that WMX-8 has a molecular weight of 938.12 and all other physicochemical properties align with theoretical expectations, and the final synthesized WMX-8 achieved a chromatographic purity of 98.75%, satisfying the quality criteria for subsequent experimental applications.

### 3.2 Cell screening and generation of IL17RA-Deficient cells

**FIGURE 2:**
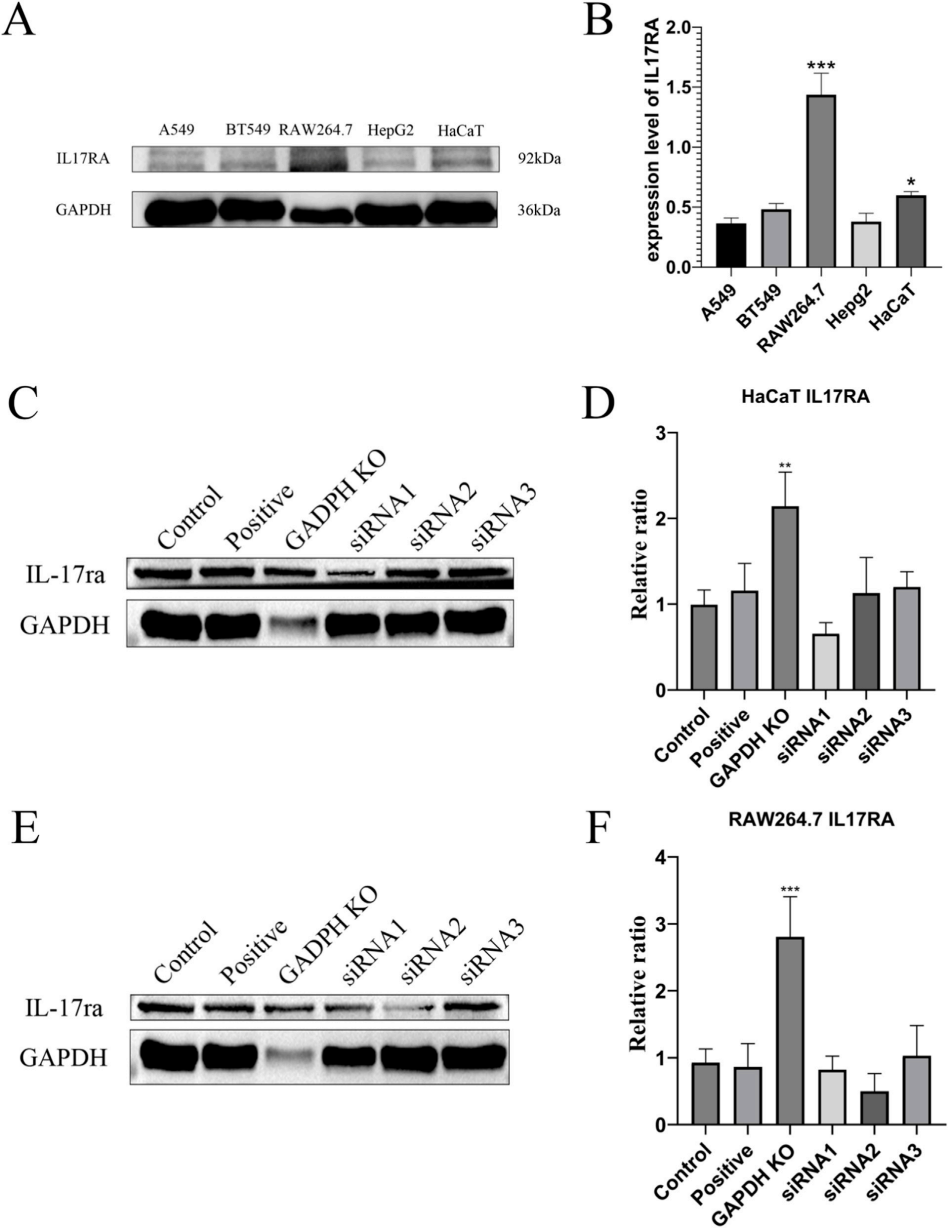
Cell screening and construction **(A)** Screening of IL17RA-highly expressing cells. **(B)** Construction of IL17RA-deficient cells. **(C–F)** Display Western blot images and corresponding quantitative analysis of gene knockdown Data are presented as mean ± standard error (SEM); statistical significance is indicated by * (P < 0.05), ** (P < 0.01), *** (P < 0.001) for intergroup differences, and ns for no significant difference (P > 0.05).

Experimental validation (Figures 2A,B) demonstrated that among the five cell lines (A549, BT549, RAW264.7, HepG2, and HaCaT), Western blot (WB) analysis revealed that RAW264.7 and HaCaT cells exhibited relatively high expression levels of IL17RA. Therefore, these two cell lines were selected as the model cells for subsequent *in vitro* experiments. Subsequently, small interfering RNA (siRNA) targeting IL17RA was transfected into these two cell lines to generate IL17RA-deficient cells, as illustrated in (Figures 2C–F), thus successfully establishing IL17RA-deficient cell models for subsequent experimental studies.

### 3.3 Analysis of targeted binding activity of WMX-8 polypeptide to IL17RA

**FIGURE 3:**
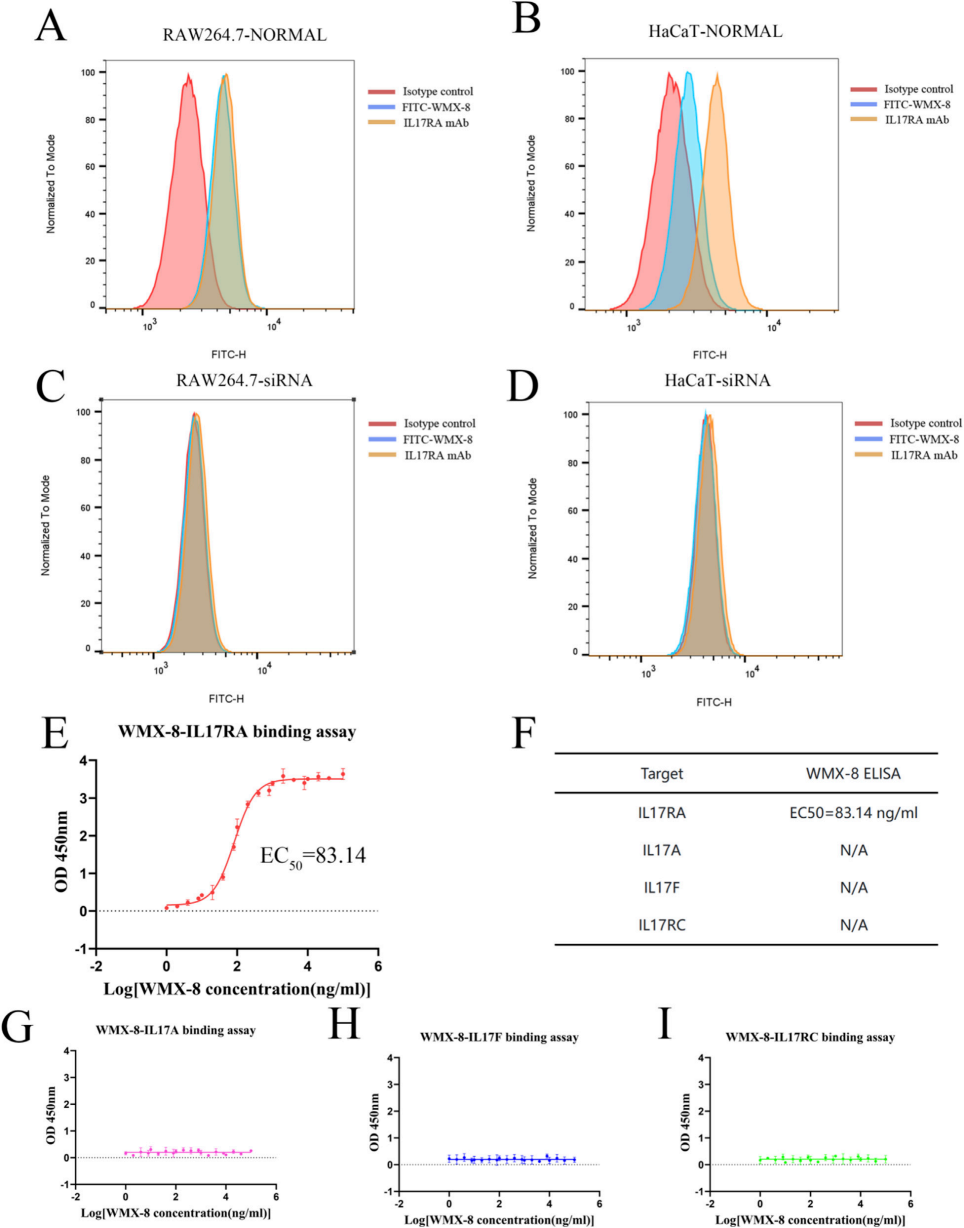
**(A–D)** Flow cytometry analysis of the binding efficiency of WMX-8 polypeptide to RAW264.7 and HaCaT cells under normal and IL17RA-knockdown conditions. **(E–I)** Enzyme-linked immunosorbent assay (ELISA) for determining the targeted binding capacity of WMX-8 polypeptide to IL17RA, the key target mediating joint inflammation.

Flow cytometry analysis demonstrated that in normal RAW264.7 and HaCaT cells (Figures 3A–D), FITC-conjugated WMX-8 polypeptide exhibited a significantly higher fluorescence signal than the isotype control, which was comparable to that of the IL17RA monoclonal antibody, indicating that WMX-8 polypeptide can directly bind to the IL17RA receptor on the cell surface. In contrast, in cells with IL17RA expression knocked down by siRNA, the fluorescence signals of both FITC-WMX-8 and IL17RA monoclonal antibody were markedly reduced and overlapped with the isotype control curve, confirming that the binding of WMX-8 polypeptide to cells is IL17RA-dependent. Collectively, these results demonstrate that WMX-8 polypeptide acts as a specific targeting ligand for IL17RA, providing direct molecular binding evidence for subsequent investigations into its anti-inflammatory effects via blocking the IL17RA pathway.

### 3.4 ELISA validates specific binding and affinity of WMX-8 polypeptide to IL17RA

ELISA binding assays demonstrated that WMX-8 polypeptide binds to IL17RA in a dose-dependent manner (Figures 3E–I), with a half-maximal effective concentration (EC_50_) of 83.14 ng/mL (Figure 3E). Notably, no significant binding was observed between WMX-8 polypeptide and IL17A, IL17F, or IL17RC (Figures 3G–I), confirming the high specificity of WMX-8 polypeptide for IL17RA. These results establish the binding affinity and molecular specificity of WMX-8 polypeptide as an IL17RA-targeting ligand, providing a critical molecular basis for further investigation of its anti-inflammatory mechanism.

### 3.5 Cytotoxicity of WMX-8 peptide

**FIGURE 4:**
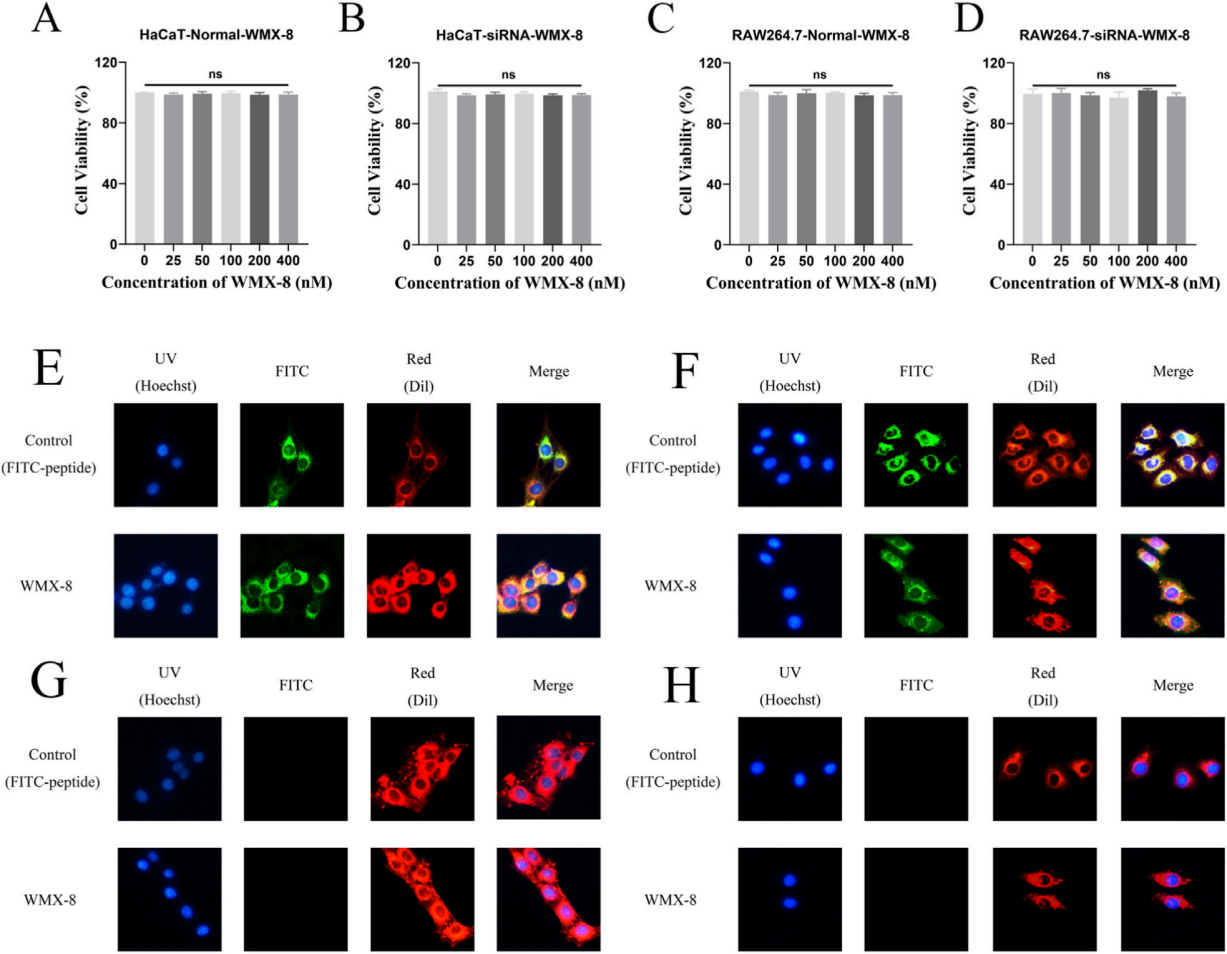
Cytotoxicity assay and binding site analysis of WMX-8 polypeptide. **(A–D)** Cytotoxicity detection of WMX-8 polypeptide in IL17RA-proficient/deficient variants of the two cell lines. **(E–H)** Immunofluorescence (IF) analysis of the binding localization and efficiency of WMX-8 polypeptide to IL17RA-proficient/deficient cells. Data are presented as mean ± standard error (SEM); statistical significance is indicated by * (P < 0.05), ** (P < 0.01), *** (P < 0.001) for intergroup differences, and ns for no significant difference (P > 0.05).

This study aimed to determine whether the WMX-8 peptide exerts direct cytotoxicity on target cells and whether such toxicity is independent of IL-17RA expression. IL-17RA-expressing cells and IL-17RA-deficient cells were co-incubated with WMX-8 peptide at various concentrations, and cell viability was assessed using the Cell Counting Kit-8 (CCK-8) assay. The results demonstrated that WMX-8 peptide did not induce cytotoxicity at any of the tested concentrations across all four cell types, indicating its favorable biosafety profile (Figures 4A–D). These findings confirm that WMX-8 peptide is non-toxic to target cells, laying a solid foundation for its application in subsequent functional studies.

### 3.6 Immunofluorescence assay validates the interaction between WMX-8 polypeptide and cells expressing or lacking IL17RA

To more intuitively visualize the binding of WMX-8 to cells and its binding sites, immunofluorescence assays were performed on both IL17RA-expressing and IL17RA-deficient variants of the two cell lines. As illustrated by the immunofluorescence results, in IL17RA-non-silenced RAW264.7 and HaCaT cells, both the FITC-labeled antibody and WMX-8-FITC specifically localized to the cell membrane region and exhibited high colocalization with the red Dil dye. This demonstrates that the tested peptide did not penetrate the cell membrane but achieved localization through specific targeting of IL17RA on the cell membrane, with no intracellular nonspecific accumulation. The fluorescence colocalization patterns among different peptide groups were essentially consistent, with no significant differences in distribution between groups (Figures 4E,F). In IL17RA-silenced cells, the green FITC fluorescence signals in the blank control group and all peptide groups were almost undetectable, without specific localization in the cell membrane region. These findings further verify that the binding of the peptide to the cell membrane is dependent on the high expression of IL17RA (Figures 4G,H).

### 3.7 Effect of WMX-8 peptide on cell motility

**FIGURE 5:**
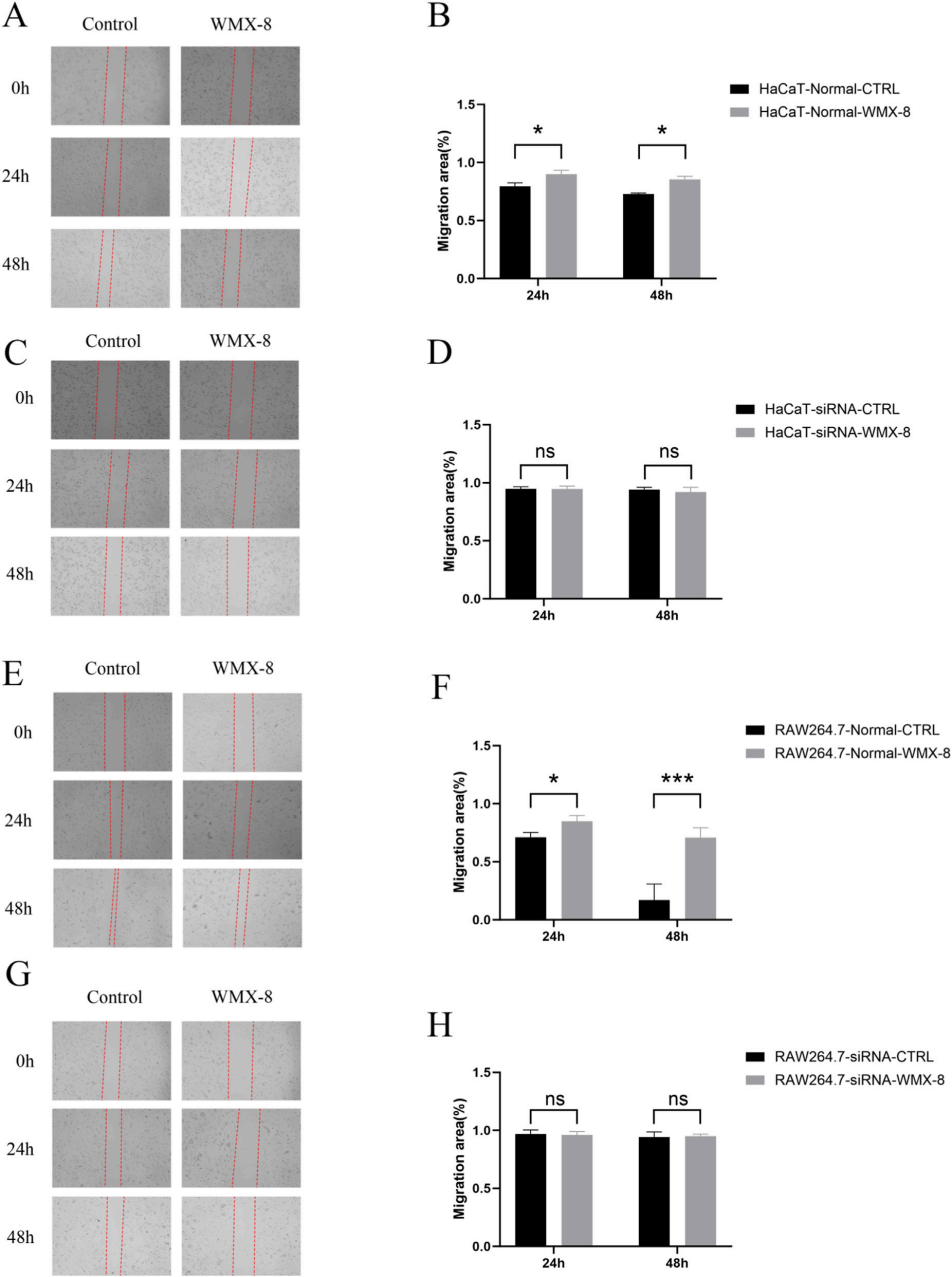
Wound healing assay was performed to evaluate the migratory ability of HaCaT and RAW264.7 cells; **(A,C,E,G)** Representative micrographs of the wound healing assay; **(B,D,F,H)** Quantitative analysis of the relative residual area ratios of scratches in the two cell lines. Data are presented as mean ± standard error (SEM); statistical significance is indicated by * (P < 0.05), ** (P < 0.01), *** (P < 0.001) for intergroup differences, and ns for no significant difference (P > 0.05).

This scratch wound healing assay was performed under serum-free conditions without exogenous IL-17A stimulation, to evaluate the effect of WMX-8 on the baseline migratory behavior of cells. The scratch wound healing assay was performed using the remaining scratch area as an indicator (a higher remaining area indicates weaker cell migration capacity). In HaCaT cells with normal IL17RA expression, the remaining scratch area of cells treated with WMX-8 polypeptide was significantly higher than that of the control group at both 24 h and 48 h (Figures 5A,B), indicating suppressed cell migration. In RAW264.7 cells with normal IL17RA expression, this inhibitory effect on cell migration was even more pronounced (Figures 5C,D), with a significantly increased remaining scratch area at 24 h and an even more obvious effect at 48 h. However, in HaCaT and RAW264.7 cells with IL17RA expression knocked down by siRNA, WMX-8 polypeptide had no significant effect on cell migration (Figures 5E–H). These results indicate that the inhibitory effect of WMX-8 polypeptide on cell migration is dependent on the expression of IL17RA on the cell surface, further confirming that WMX-8 polypeptide exerts its biological functions by specifically targeting IL17RA.

### 3.8 *In vitro* inflammatory modulation and therapeutic effects of WMX-8 peptide targeting the IL17RA signaling pathway

**FIGURE 6:**
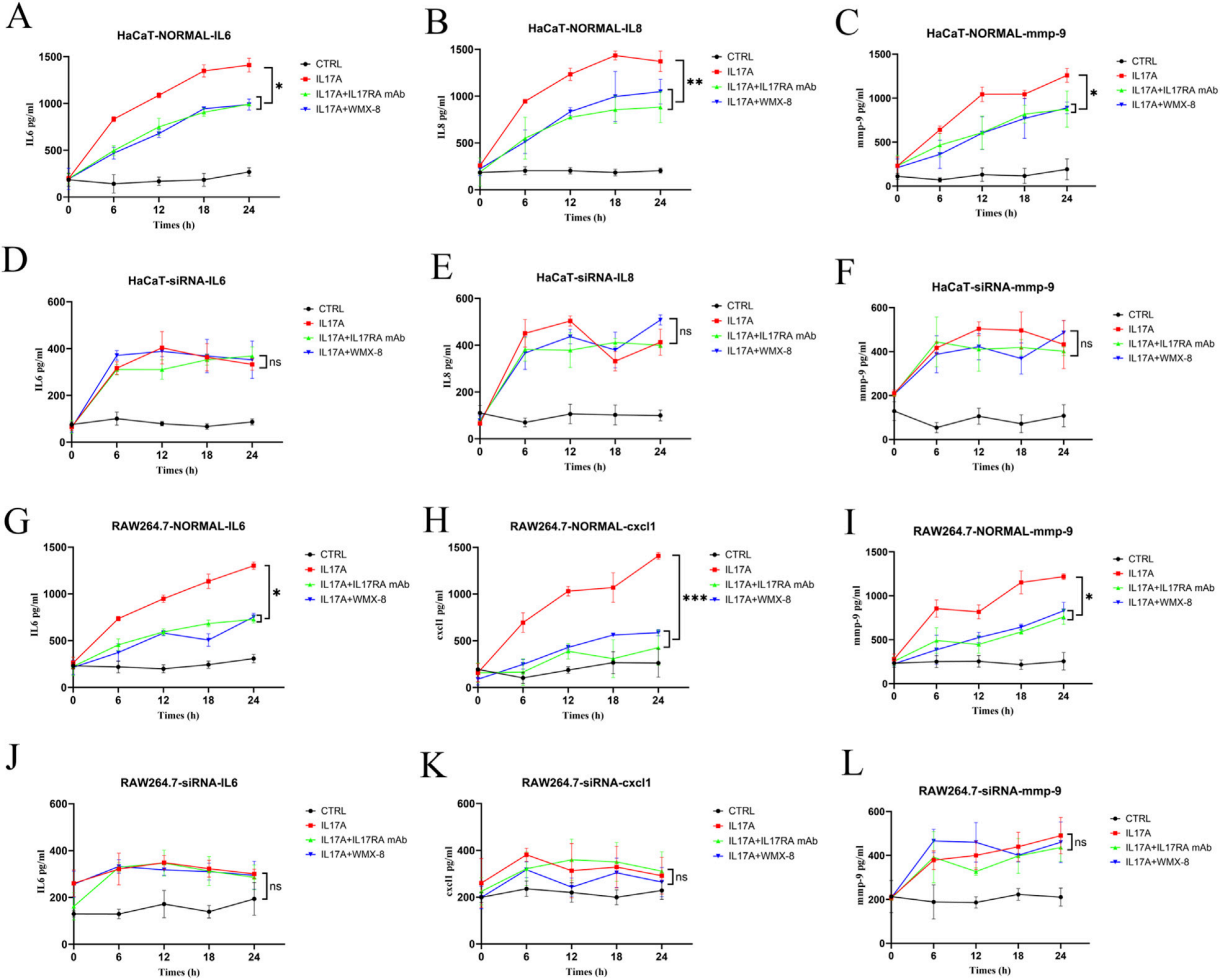
Analysis of dynamic changes in pro-inflammatory cytokine secretion from various cells under different WMX-8 treatments by ELISA. **(A–F)** Changes in pro-inflammatory cytokine concentrations over time (0–24 h) in HaCaT cells (IL17RA-normal expression subtype (NORMAL) or siRNA-mediated IL17RA-deficient subtype (siRNA)) after different treatments, detected by ELISA: control group (CTRL), IL17A stimulation group, IL17A combined with IL17RA monoclonal antibody group (IL17A + IL17RA mAb), and IL17A combined with WMX-8 peptide group (IL17A + WMX-8). Among these, **(A–C)** correspond to the NORMAL subgroup of HaCaT cells, detecting the concentrations of IL-6, IL-8, and MMP-9, respectively; **(D–F)** correspond to the IL17RA-siRNA subgroup of HaCaT cells, with the same detection indicators as above. **(G–L)** Changes in pro-inflammatory cytokine concentrations over time (0–24 h) in RAW264.7 cells (IL17RA-normal expression subtype (NORMAL) or siRNA-mediated IL17RA-deficient subtype (siRNA)) after the above four treatments, detected by ELISA. Among these, **(G–I)** correspond to the NORMAL subgroup of RAW264.7 cells, detecting the concentrations of IL-6, CXCL1, and MMP-9, respectively; **(J–L)** correspond to the IL17RA-siRNA subgroup of RAW264.7 cells, with the same detection indicators as above. The ordinate of each subplot represents the concentration of the corresponding cytokine (unit: pg/mL), and the abscissa represents the culture time (unit: h). Experimental data are presented as mean ± standard error (SEM); statistical significance is indicated by * (P < 0.05), ** (P < 0.01), *** (P < 0.001) for intergroup differences, and ns for no significant difference (P > 0.05).

To clarify the regulatory role of the WMX-8 peptide in IL17RA-mediated inflammatory responses, combined quantitative real-time polymerase chain reaction (qPCR, transcriptional level) and Enzyme-Linked Immunosorbent Assay (ELISA, protein secretion level) analyses were performed to systematically evaluate its effects in IL17RA-highly expressing wild-type and siRNA-mediated IL17RA-silenced deficient HaCaT and RAW264.7 cells. The qPCR results showed that in IL17RA-highly expressing wild-type HaCaT and RAW264.7 cells, the mRNA transcription levels of various pro-inflammatory cytokines were significantly increased after IL17A stimulation; however, following treatment with the WMX-8 peptide, the mRNA expression levels of these pro-inflammatory cytokines were significantly inhibited, and the inhibitory effect was comparable to that of the IL17RA monoclonal antibody (IL17RA mAb) positive control group. In contrast, no significant difference in the mRNA expression levels of pro-inflammatory cytokines was observed between the WMX-8 peptide-treated group and the control group in the two IL17RA-deficient cell lines (Supplementary Figure S1). The ELISA experiment further verified the above results at the protein secretion level: in wild-type HaCaT and RAW264.7 cells, the protein secretion of pro-inflammatory cytokines such as IL-6, TNF-α, CXCL11, and MMP-9 was significantly increased after IL17A stimulation; whereas WMX-8 peptide treatment effectively downregulated the protein release levels of these pro-inflammatory cytokines, and this inhibitory effect was time-dependent, consistent with the action trend of IL17RA mAb. In IL17RA-deficient cells, the WMX-8 peptide had no obvious regulatory effect on the protein secretion of pro-inflammatory cytokines, which was highly consistent with the qPCR results (Figure 6). Collectively, the WMX-8 peptide can specifically inhibit IL17A-induced pro-inflammatory cytokine production through targeting the IL17RA signaling pathway at two key stages: transcription and protein secretion. Its anti-inflammatory effect is strictly dependent on the expression of IL17RA on the cell surface, and it exhibits similar targeted regulatory activity to IL17RA mAb, providing solid *in vitro* experimental data support for the targeted therapy of IL17RA-mediated inflammatory diseases.

### 3.9 *In vivo* inflammatory modulation and therapeutic effects of WMX-8 peptide targeting the IL17RA signaling pathway

**FIGURE 7:**
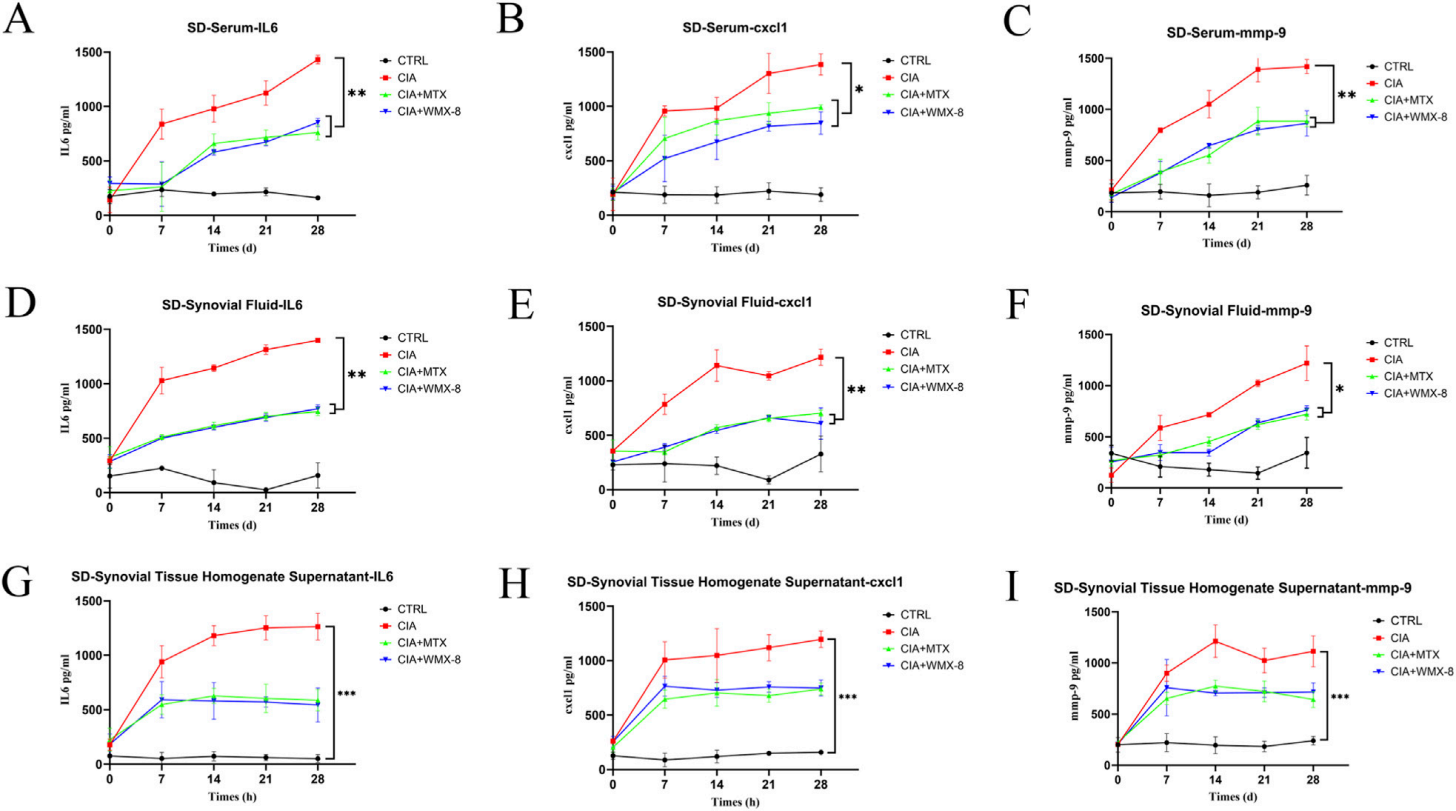
Dynamic analysis of pro-inflammatory cytokine protein expression in collagen-induced arthritis (CIA) model SD rats under different treatments (Enzyme-linked immunosorbent assay, ELISA). **(A–I)** ELISA was performed to detect the dynamic changes in pro-inflammatory cytokine concentrations in serum, ankle joint cavity fluid, and synovial tissue homogenate supernatant of SD rats from the control group (CTRL), collagen-induced arthritis group (CIA), CIA + methotrexate (MTX) treatment group (CIA + MTX), and CIA + WMX-8 polypeptide group (CIA + WMX-8) at 0–28 days post-modeling. Among them, **(A–C)** show the concentration detection results of IL-6, CXCL1, and MMP-9 in serum samples; **(D–F)** show the results in ankle joint cavity fluid samples (the same cytokines as those in serum); **(G–I)** show the results in synovial tissue homogenate supernatant samples (the same cytokines as those in serum). For each ELISA subfigure, the ordinate represents the concentration of the corresponding cytokine (unit: pg/mL), and the abscissa represents the days post-modeling (unit: days). Data are presented as mean ± standard error (SEM); statistical significance is indicated by * (P < 0.05), ** (P < 0.01), *** (P < 0.001) for intergroup differences, and ns for no significant difference (P > 0.05).

The *in vivo* experimental results of Collagen-Induced Arthritis (CIA) model Sprague-Dawley (SD) rats demonstrated that, compared with the control group, the levels of CXCL1 in serum, IL-6, CXCL1 and matrix metalloproteinase (MMP) proteins in ankle joint cavity fluid, as well as the expressions of IL-6 and CXCL1 in synovial tissue homogenate supernatant were significantly increased in the CIA model group (Figure 7) (Supplementary Figure S2). Whereas after treatment with the WMX-8 peptide, the expression levels of the aforementioned pro-inflammatory cytokines (IL-6, CXCL1) and MMP were significantly suppressed, and its regulatory effect was consistent with that of the MTX intervention group. Taken together with the qPCR and ELISA results, the WMX-8 peptide can effectively inhibit local and systemic inflammatory responses in CIA model rats by targeting the IL17RA signaling pathway *in vivo*, reducing the production of pro-inflammatory cytokines and damage-related proteases in joint tissues. This provides reliable *in vivo* experimental evidence for the WMX-8 peptide as a potential therapeutic agent for IL17RA-mediated arthritis.

### 3.10 Effect of WMX-8 peptide on joint range of motion (ROM) in Sprague-Dawley (SD) rats

**FIGURE 8:**
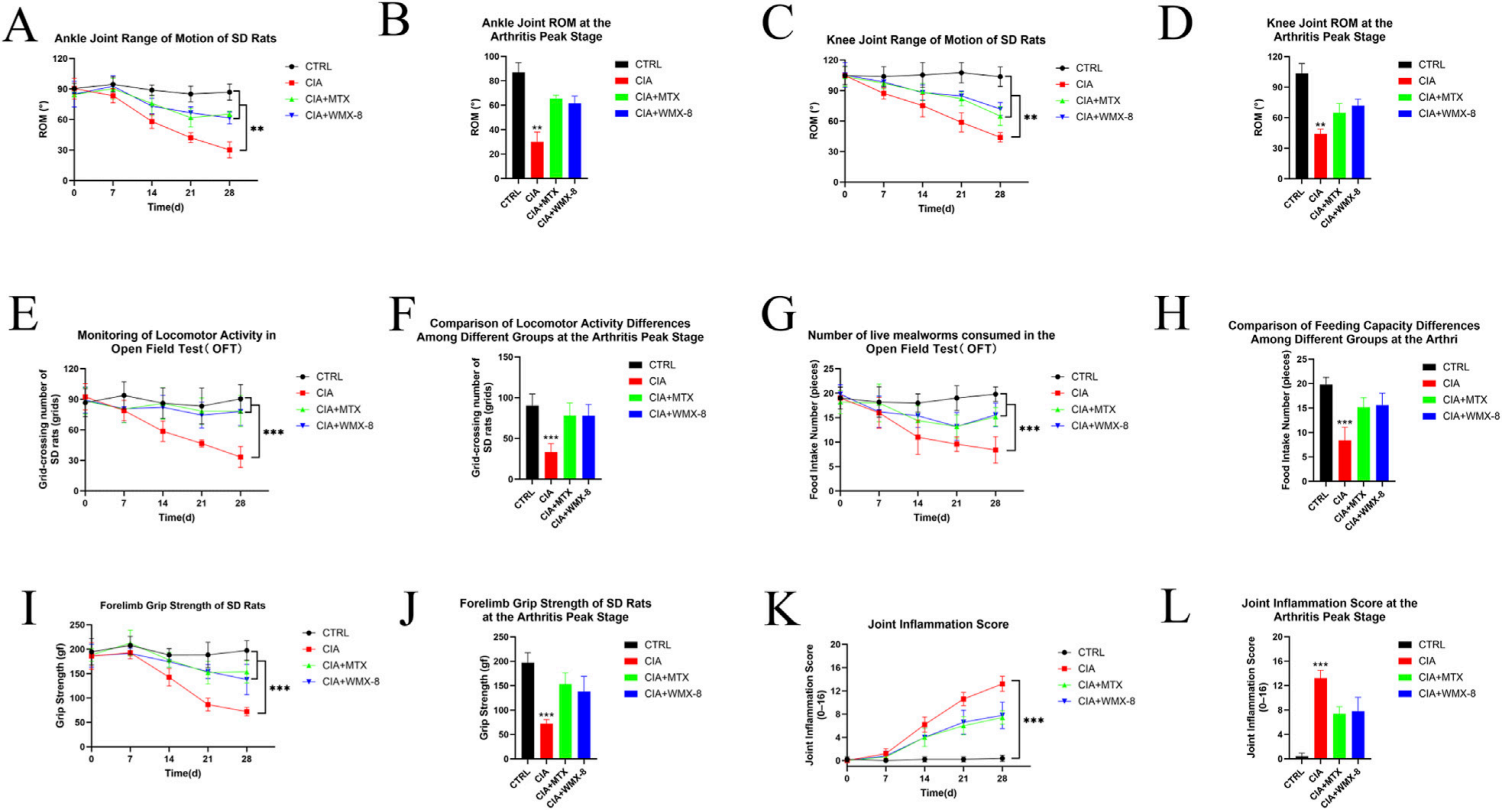
Multi-index dynamic monitoring of joint function, locomotor ability and inflammatory status in SD rat CIA model. **(A–D)** Dynamic changes in ankle and knee joint range of motion (ROM) and intergroup comparison at the arthritis peak stage in SD rats under different treatments: control group (CTRL), collagen-induced arthritis group (CIA), CIA + methotrexate (MTX) treatment group (CIA + MTX), and CIA + WMX-8 peptide group (CIA + WMX-8). Among these, **(A,B)** represent ankle joint ROM **(A)** dynamic changes from 0 to 28 days; **(B)** intergroup comparison at the arthritis peak stage); **(C,D)** represent knee joint ROM (C: dynamic changes from 0 to 28 days; **(D)** intergroup comparison at the arthritis peak stage). **(E–H)** Dynamic changes in locomotor ability of SD rats detected by Open Field Test (OFT) and intergroup comparison at the arthritis peak stage; **(E,F)** represent grid-crossing number **(E)** dynamic changes from 0 to 28 days; **(F)** intergroup comparison at the arthritis peak stage); **(G,H)** represent food intake number **(G)** dynamic changes from 0 to 28 days; **(H)** intergroup comparison at the arthritis peak stage). **(I,J)** Dynamic changes in forelimb grip strength of SD rats under different treatments and intergroup comparison at the arthritis peak stage **(L)** dynamic changes from 0 to 28 days; **(J)** intergroup comparison at the arthritis peak stage). **(K,L)** Dynamic changes in joint inflammation score of SD rats under different treatments and intergroup comparison at the arthritis peak stage (**(K)** dynamic changes from 0 to 28 days; **(L)** intergroup comparison at the arthritis peak stage). The abscissa of dynamic monitoring subplots represents days after modeling (unit: day), and the ordinate represents the detection value of the corresponding index; intergroup comparison subplots represent the statistical values of indicators at the arthritis peak stage. Experimental data are presented as mean ± standard error (SEM); statistical significance is indicated by * (P < 0.05), ** (P < 0.01), *** (P < 0.001) for intergroup differences, and ns for no significant difference (P > 0.05).

To assess the impact of the WMX-8 peptide on ankle and knee joint range of motion (ROM) in Sprague-Dawley (SD) rats with collagen-induced arthritis (CIA), the present study conducted corresponding measurements, and the results demonstrated that: The ankle and knee ROM of rats in the control group (CTRL) remained stable throughout the 28-day experimental period; in contrast, the ankle and knee ROM of rats in the CIA model group decreased significantly as the modeling duration prolonged (Figures 8A,C), and at the peak stage of arthritis, their ankle and knee ROM were markedly reduced compared with those in the CTRL group (Figures 8B,D). Following treatment with the WMX-8 peptide, the decrease in ankle and knee ROM of CIA rats was significantly attenuated. At the peak stage of arthritis, the ankle and knee ROM of WMX-8-treated CIA rats were notably higher than those in the CIA model group, and this improvement effect was comparable to that of the MTX positive control group (CIA + MTX). Collectively, the WMX-8 peptide can effectively improve the ankle and knee ROM of CIA model SD rats, alleviating joint dysfunction caused by arthritis.

### 3.11 Monitoring of the effect of WMX-8 peptide on locomotor activity in rats

Using the Open Field Test (OFT), with the grid-crossing number (reflecting spontaneous activity level) and food-initiated number (reflecting foraging enthusiasm) as indicators, we monitored the effect of the WMX-8 peptide on the locomotor activity of collagen-induced arthritis (CIA) model Sprague-Dawley (SD) rats. The results showed that: the grid-crossing number and food-initiated number of rats in the control group (CTRL) remained stable throughout the 28-day experimental period (Figures 8E,G); in contrast, the grid-crossing number of rats in the CIA model group decreased significantly as the modeling duration prolonged, and the food-initiated number also showed a marked downward trend. At the peak stage of arthritis, both the grid-crossing number and food-initiated number of the CIA group were extremely significantly lower than those of the CTRL group (Figures 8F,H). After treatment with the WMX-8 peptide, the decrease in the grid-crossing number of CIA rats was significantly attenuated, and the reduction in the food-initiated number was also effectively alleviated; at the peak stage of arthritis, both of the above indicators in the CIA + WMX-8 group were significantly higher than those in the CIA model group, and this improvement effect was comparable to that of the MTX positive control group (CIA + MTX). Collectively, the WMX-8 peptide can effectively improve the spontaneous activity and foraging enthusiasm of CIA model SD rats, alleviating the decline in overall locomotor activity caused by arthritis.

### 3.12 Effect of WMX-8 peptide on forelimb grip strength in collagen-induced arthritis (CIA) model Sprague-Dawley (SD)

To clarify the potential of the WMX-8 peptide to improve motor function in Sprague-Dawley (SD) rats with collagen-induced arthritis (CIA), this study employed the forelimb grip strength test to evaluate its regulatory effect on the grip function of model rats. The results showed that: the forelimb grip strength of rats in the control group (CTRL) remained at a stable baseline level throughout the 28-day experimental period (Figure 8I); in contrast, the forelimb grip strength of CIA model rats exhibited a continuous downward trend as the arthritis progressed, and by the peak stage of arthritis, their grip strength was extremely significantly lower than that of the CTRL group (Figure 8J). Following intervention with the WMX-8 peptide, the downward trend in forelimb grip strength of CIA model rats was significantly suppressed; at the peak stage of arthritis, the grip strength level of the CIA + WMX-8 group was significantly higher than that of the CIA model group, and this improvement effect was comparable to the regulatory effect of the MTX positive control group (CIA + MTX). These results indicate that the WMX-8 peptide can effectively enhance the forelimb grip strength of CIA model SD rats, thereby alleviating the motor function impairment caused by arthritis.

### 3.13 Modulatory effect of WMX-8 peptide on arthritis score in collagen-induced arthritis (CIA) model SD rats

The WMX-8 peptide can effectively reduce the joint inflammation score of Sprague-Dawley (SD) rats with collagen-induced arthritis (CIA), alleviating the local inflammatory response caused by arthritis. In this experiment, the joint inflammation score was used to evaluate the improvement effect of the WMX-8 peptide on the severity of joint inflammation in CIA model SD rats. The results showed that: the joint inflammation score of rats in the control group (CTRL) remained at a stable low level throughout the 28-day experimental period (Figure 8K); in contrast, the joint inflammation score of CIA model rats showed a significant upward trend as the modeling duration prolonged, and at the peak stage of arthritis, their score was extremely significantly higher than that of the CTRL group (Figure 8L). After treatment with the WMX-8 peptide, the increase in the joint inflammation score of CIA rats was significantly suppressed; at the peak stage of arthritis, the joint inflammation score of the CIA + WMX-8 group was significantly lower than that of the CIA model group, and its anti-inflammatory effect was comparable to that of the MTX positive control group (CIA + MTX).

### 3.14 Dynamic alleviatory effect of WMX-8 peptide on ankle and knee joint swelling in collagen-induced arthritis (CIA) model SD rats

In this experiment, we investigated the interventional effect of the WMX-8 peptide on joint swelling in collagen-induced arthritis (CIA) model Sprague-Dawley (SD) rats by dynamically monitoring the ankle and knee joint circumferences (used as an indicator of swelling severity), while integrating the trend changes in line charts and the color scale distribution in heatmaps.

**FIGURE 9:**
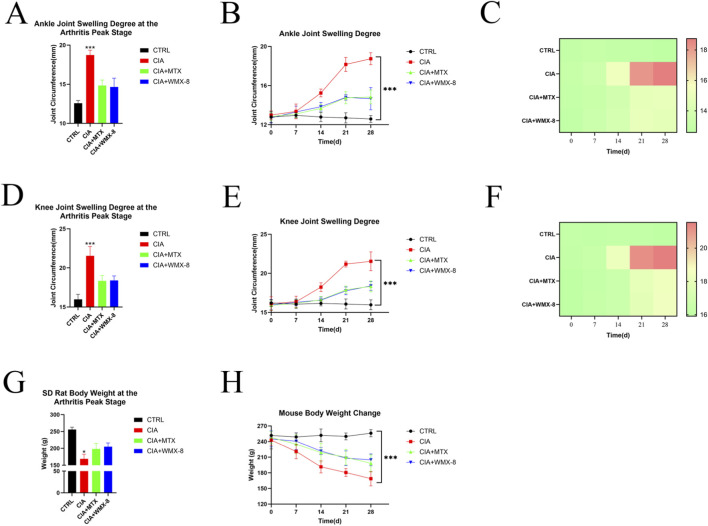
Dynamic monitoring and intergroup comparison of joint swelling and body weight changes in SD rat CIA model. **(A–C)** Detection of ankle joint swelling in SD rats under different treatments: control group (CTRL), collagen-induced arthritis group (CIA), CIA + methotrexate (MTX) treatment group (CIA + MTX), and CIA + WMX-8 peptide group (CIA + WMX-8). Among these, **(A)** represents intergroup comparison of ankle joint circumference at the arthritis peak stage; **(B)** represents dynamic changes in ankle joint circumference from 0 to 28 days; **(C)** represents heatmap analysis of ankle joint swelling at different time points. **(D–F)** Detection of knee joint swelling in SD rats under different treatments: **(D)** represents intergroup comparison of knee joint circumference at the arthritis peak stage; **(E)** represents dynamic changes in knee joint circumference from 0 to 28 days; **(F)** represents heatmap analysis of knee joint swelling at different time points. **(G,H)** Changes in animal body weight under different treatments: **(G)** represents intergroup comparison of body weight in SD rats at the arthritis peak stage; **(H)** represents dynamic changes in body weight in rat from 0 to 28 days. The abscissa of dynamic monitoring subplots represents days after modeling (unit: day), and the ordinate represents the detection value of the corresponding index (joint circumference: mm, body weight: g); the color of the heatmap represents the relative level of swelling or body weight. Experimental data are presented as mean ± standard error (SEM); statistical significance is indicated by * (P < 0.05), ** (P < 0.01), *** (P < 0.001) for intergroup differences, and ns for no significant difference (P > 0.05).

The results showed that: the ankle and knee joint circumferences of rats in the control group (CTRL) remained at a stable baseline level throughout the 28-day experimental period, and the color scale of the corresponding heatmap also stayed in the low range (Figures 9C,F). In contrast, the ankle and knee joint circumferences of the CIA model group exhibited a marked upward trend as the arthritis progressed, and by the peak stage of arthritis, their circumferences were extremely significantly higher than those of the CTRL group (Figures 9A,D). The color scale of the corresponding period in the heatmap deepened significantly, which intuitively reflected the continuous aggravation of swelling severity.

Following intervention with the WMX-8 peptide, the rising rate of the ankle and knee joint circumferences in CIA rats was significantly slowed down (Figures 9B,E), and the magnitude of color scale change in the heatmap for this group was much smaller than that of the CIA model group. At the peak stage of arthritis, the ankle and knee joint circumferences of the CIA + WMX-8 group were significantly lower than those of the CIA model group, and this alleviating effect was comparable to the interventional effect of the MTX positive control group.

These results indicate that the WMX-8 peptide does not only ameliorate swelling at the peak stage of inflammation, but rather continuously alleviates the swelling severity of the ankle and knee joints in CIA model SD rats by regulating the dynamic change trend of joint circumferences during the disease course, providing intuitive morphological evidence for its improvement of the local pathological state of arthritis.

### 3.15 Ameliorative effect of WMX-8 peptide on body weight changes in collagen-induced arthritis (CIA) model Sprague-Dawley (SD) rats

In this experiment, body weight changes were used as a core indicator reflecting the overall health status of the organism to investigate the regulatory effect of the WMX-8 peptide on the body weight of CIA model SD rats. The results showed that: the body weight of rats in the control group (CTRL) remained at a stable baseline level throughout the 28-day experimental period; in contrast, the body weight of CIA model rats exhibited a continuous downward trend as the arthritis progressed, and by the peak stage of arthritis, their body weight was significantly lower than that of the CTRL group (Figure 9G). Following intervention with the WMX-8 peptide, the downward trend in body weight of CIA rats was significantly curbed (Figure 9H); at the peak stage of arthritis, the body weight of the CIA + WMX-8 group was significantly higher than that of the CIA model group, and this ameliorative effect was comparable to the MTX positive control group (CIA + MTX). These results indicate that the WMX-8 peptide can effectively alleviate the body weight loss of CIA model SD rats, promoting the recovery of their overall health status.

### 3.16 HE staining conclusion of WMX-8 polypeptide on collagen-induced arthritis (CIA) model SD rats

**FIGURE 10:**
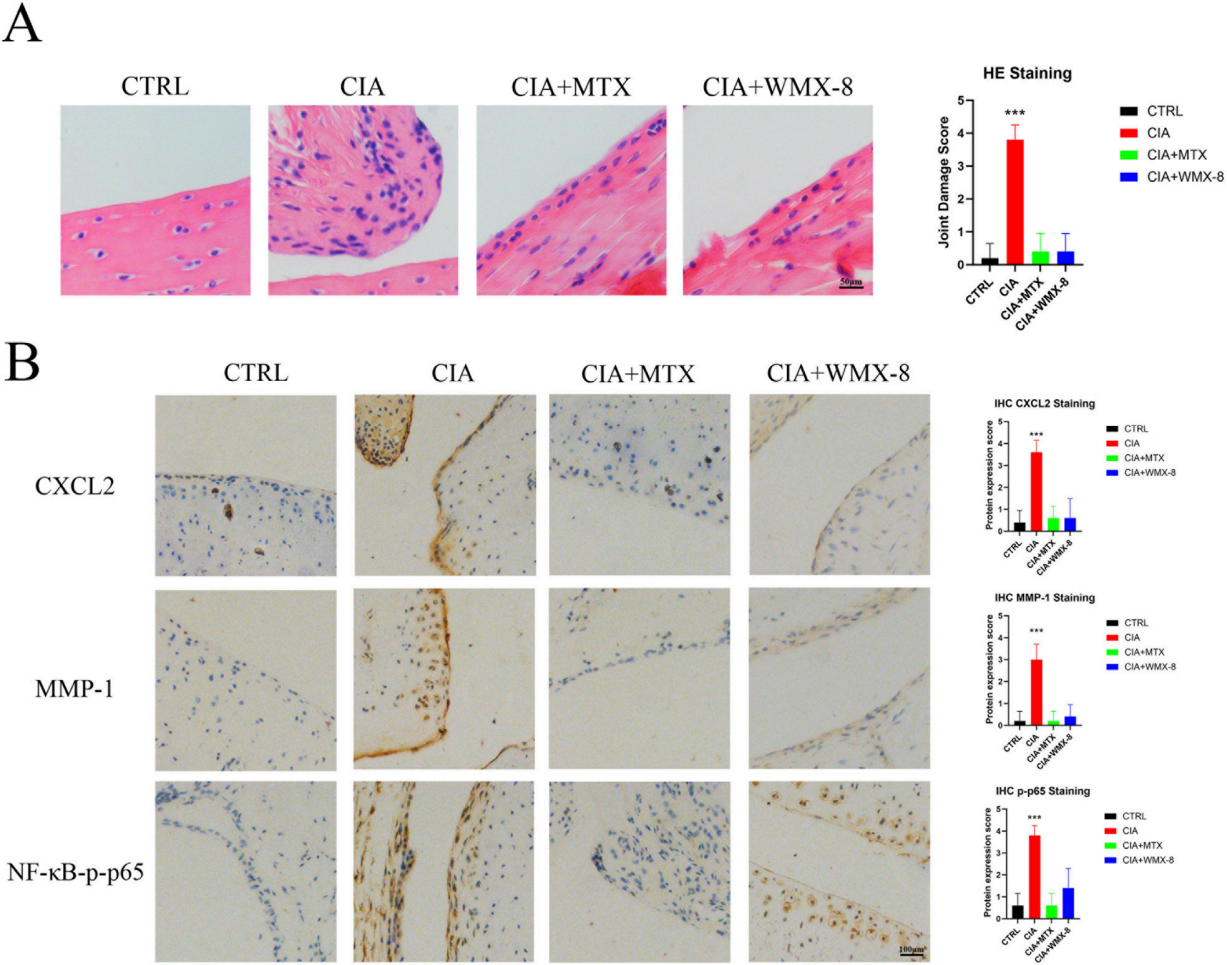
Effects of different interventions on pathological damage and pro-inflammatory factor expression in synovial tissue of CIA model rats, **(A)** Hematoxylin-eosin (HE) staining was used to observe the pathological morphology and inflammation scores of synovial tissue in the control group (CTRL), collagen-induced arthritis group (CIA), CIA + methotrexate (MTX) treatment group (CIA + MTX), and CIA + WMX-8 polypeptide treatment group (CIA + WMX-8). **(B)** Immunohistochemistry (IHC) staining was performed to detect the protein expression and localization (brownish-yellow staining indicates positive expression) of CXCL2, MMP-1, and NF-κB-p65 in synovial tissue of each group, as well as the corresponding protein expression level scores. Experimental data are presented as mean ± standard error (SEM); statistical significance is indicated by * (P < 0.05), ** (P < 0.01), *** (P < 0.001) for intergroup differences, and ns for no significant difference (P > 0.05).

HE staining results demonstrated that synovial tissues from the control group (CTRL) exhibited normal morphology with neatly arranged cells, consisting of only 1–2 layers of flat cells, and no inflammatory cell infiltration or tissue edema. In contrast, synovial tissues from the collagen-induced arthritis (CIA) group showed obvious pathological damage, characterized by significant synovial thickening, increased cell layers, massive inflammatory cell infiltration, and tissue edema. Importantly, synovial hyperplasia and inflammatory cell infiltration were significantly alleviated in both the CIA + methotrexate (MTX) treatment group (CIA + MTX) and CIA + WMX-8 polypeptide treatment group (CIA + WMX-8), with synovial morphology approaching that of the control group. These findings indicate that both anti-IL17RA antibody and WMX-8 polypeptide can effectively alleviate CIA-induced synovial pathological damage (Figure 10A).

### 3.17 IHC staining conclusion of WMX-8 polypeptide on collagen-induced arthritis (CIA) model SD rats

Consistent with the HE staining findings, IHC analysis revealed minimal positive staining for CXCL2, MMP-1, and total NF-κB p65 in the control (CTRL) group.

In stark contrast, intense staining of these targets was detected in the cytoplasm and nuclei of synovial cells from the collagen-induced arthritis (CIA) group. Notably, both the CIA + methotrexate (MTX) and CIA + WMX-8 polypeptide treatment groups exhibited substantially attenuated positive staining intensity of the above proteins. These phenotypic observations suggest that IL17RA blockade by antibody or WMX-8 may be linked to altered expression or distribution of NF-κB p65, as well as reduced levels of pro-inflammatory mediators (CXCL2 and MMP-1), which contributes to alleviated synovial inflammation and tissue damage in the CIA model. Direct verification of NF-κB pathway activation requires detection of phosphorylated p65 and IκBα, which was not performed in the present study (Figure 10B).

## 4 Discussion

This study systematically explored the anti-inflammatory activity and therapeutic potential of the WMX-8 peptide targeting the IL17RA pathway through a comprehensive research framework covering peptide design, *in vitro* cellular experiments, and *in vivo* animal validation. The core findings demonstrated that WMX-8 peptide exerts dual regulatory effects on inflammatory responses and cellular functions in both IL17RA-expressing cells and collagen-induced arthritis (CIA) models of SD rats, with its biological activity strictly dependent on IL17RA expression.

*In vitro* experiments (Figures 1–6) revealed that WMX-8 peptide, after rational design and synthesis, specifically inhibited IL17A-induced pro-inflammatory responses in HaCaT and RAW264.7 cells. ELISA and QPCR results consistently showed that WMX-8 peptide downregulated the mRNA and protein expression levels of IL-6, CXCL1, and MMP-9, which are key mediators of inflammatory cascades and tissue remodeling. Notably, this inhibitory effect was almost completely abrogated in IL17RA-deficient cells constructed by siRNA transfection (Figure 2), confirming that WMX-8 peptide’s anti-inflammatory activity is mediated through specific targeting of IL17RA rather than non-specific cytotoxicity—supported by CCK8 assays showing no significant impact on cell viability (Figure 4). Furthermore, scratch assay results indicated that WMX-8 peptide suppressed cellular migration (Figure 5), which aligns with previous findings that the IL17RA pathway promotes cell migration by upregulating MMP family members. It is worth noting that this inhibitory effect on cell migration was evaluated under unstimulated conditions, reflecting modulation of the basal signaling activity of IL17RA. As a competitive antagonist targeting IL17RA, WMX-8 can bind to the receptor even in the absence of exogenous IL-17A, thereby inhibiting its constitutive signaling and reducing the baseline migratory behavior of cells. Consistently, the downregulation of MMP-9 induced by WMX-8 further explains its anti-migratory effect, since MMP-9 is a well-recognized key driver of cell motility. The complete loss of this effect in IL17RA-knockdown cells confirms its specificity, which is fully consistent with the proposed antagonistic mechanism of WMX-8. Collectively, these data suggest that WMX-8 peptide likely functions as a competitive antagonist, binding to the ligand-binding domain of IL17RA to block IL17A-IL17RA interaction. This action may modulate downstream signaling cascades including the NF-κB and p38MAPK pathways, and further reduce the expression of pro-inflammatory cytokines and migration-related molecules.

*In vivo* validation using SD rat CIA models (Figures 7–9) further confirmed the biological activity of WMX-8 peptide. WMX-8 treatment significantly alleviated joint swelling (ankle and knee circumference reduction), decreased arthritis scores, and improved motor function (enhanced grid-crossing ability in open field tests) and weight recovery in CIA rats. These improvements were accompanied by reduced pro-inflammatory cytokine levels in serum, ankle joint cavity fluid, and synovial tissues, consistent with *in vitro* findings. Consistent results across multiple tissues and detection indexes show that WMX-8 produces robust systemic anti-inflammatory effects and alleviates pathological damage in CIA rats.

Current therapeutic strategies targeting the IL17RA pathway mainly rely on monoclonal antibodies such as Secukinumab, which exhibit significant clinical efficacy but suffer from limitations including high production costs, strong immunogenicity, and the need for intravenous administration. Methotrexate (MTX), the first-line conventional drug for rheumatoid arthritis, was used as the positive control in our *in vivo* CIA model. Although a few IL17RA-targeting peptides (e.g., AL-8 (0)) have been reported, they lack systematic *in vivo* validation and comprehensive functional evaluation. The innovation of this study lies in three key aspects: First, it establishes a complete research chain from rational peptide design (Figure 1) to IL17RA-deficient cell validation (Figure 2), and further to functional verification (inflammatory regulation, migration inhibition) and *in vivo* therapeutic assessment (Figures 7–9), providing rigorous evidence for WMX-8 peptide’s specificity and efficacy. Second, unlike previous studies focusing solely on inflammatory cytokine expression, this work simultaneously evaluated cellular functional changes (viability, migration) and multiple *in vivo* indicators (joint function, motor ability, weight), comprehensively reflecting the peptide’s multi-dimensional regulatory effects. Third, the consistent validation across HaCaT and RAW264.7 macrophages (Figures 3–6) demonstrates that WMX-8 peptide’s anti-inflammatory activity is not cell-type-specific, expanding its potential application scenarios beyond rheumatoid arthritis to other IL17RA-associated inflammatory diseases.

Notably, the regulatory effect of WMX-8 peptide on MMP-9 expression (Figures 5, 6) holds particular significance. MMP-9 is a key matrix metalloproteinase involved in synovial tissue invasion and cartilage destruction in arthritis, and its overexpression is closely associated with IL17RA pathway activation. By inhibiting MMP-9 at both transcriptional and translational levels, WMX-8 peptide not only alleviates acute inflammation but also potentially mitigates long-term joint tissue damage—an advantage over some anti-inflammatory drugs that only target cytokine secretion without affecting tissue remodeling processes.

Despite the compelling findings, this study has several limitations that require attention. First, the *in vitro* experiments employed established cell lines (HaCaT and RAW264.7) rather than primary cells such as synovial macrophages or articular chondrocytes, which may differ in biological characteristics from native inflammatory microenvironment cells. Second, the *in vivo* validation was restricted to the CIA model, which primarily mimics rheumatoid arthritis; the therapeutic efficacy of WMX-8 peptide in other arthritis models (e.g., osteoarthritis, gouty arthritis) remains untested. Third, critical pharmacodynamic and safety data are lacking. The relative competitive binding activity of WMX-8 against IL17RA was evaluated by competitive ELISA, which is an indirect detection method and may be interfered by plate adsorption artifacts. Although we have set up multiple control groups and optimized experimental operations to minimize such non-specific interference, the inherent drawbacks of ELISA cannot be fully eliminated. The EC50 values obtained from this assay cannot be equated to the direct binding affinity or dissociation constant (Kd) of the peptide. Direct verification of binding characteristics and specific binding sites relies on surface plasmon resonance (SPR) or microscale thermophoresis (MST), which are currently unavailable in our local research platforms. Besides the binding profile, pharmacokinetic profiles and long-term toxicity assessments are also absent—these are all essential prerequisites for clinical translation. In our follow-up studies, we will actively cooperate with external public research platforms and strive to carry out SPR and MST experiments to further clarify the exact binding parameters and interaction sites between WMX-8 and IL17RA.Finally, the study did not investigate the downstream signaling molecules (e.g., phosphorylated p38MAPK) affected by WMX-8 peptide, which would help clarify the precise molecular mechanism of IL17RA pathway inhibition.

Furthermore, although we confirmed the loss of WMX-8 activity in IL17RA-deficient cells generated by siRNA interference, we did not perform IL17RA rescue experiments to fully rule out potential off-target effects of siRNA. The siRNA used in this study has been well validated in previous literature and showed no obvious cytotoxicity to cells. Additionally, the phenomenon that WMX-8 lost efficacy was consistent across multiple functional assays including cytokine secretion and cell migration, which collectively supports the specific IL17RA-dependent mechanism of WMX-8. Nevertheless, rescue experiments with IL17RA re-expression are still needed to provide definitive causal evidence, and we will carry out this verification in our follow-up research.

To address these limitations, future studies should focus on the following directions: First, validate the anti-inflammatory activity of WMX-8 peptide in primary synovial macrophages and chondrocytes isolated from CIA models to enhance clinical relevance. Second, extend the research to other inflammatory disease models to evaluate the peptide’s broad-spectrum anti-inflammatory potential. Third, use molecular docking, surface plasmon resonance, and site-directed mutagenesis to identify the specific binding domain between WMX-8 peptide and IL17RA, providing a structural basis for peptide optimization. Fourth, conduct in-depth mechanistic studies to assess the regulatory effects of WMX-8 peptide on key signaling molecules such as p38MAPK and NF-κB in the IL17RA pathway. Finally, perform systematic pharmacokinetic studies to determine the optimal administration route, dosage, and treatment course, and evaluate long-term toxicity and immunogenicity to lay the foundation for clinical application.

In summary, this study demonstrates that the WMX-8 peptide exerts potent anti-inflammatory effects and improves joint function by specifically targeting the IL17RA pathway, with consistent efficacy verified in both *in vitro* cellular models and *in vivo* CIA models. In our animal experiments, the therapeutic efficacy of WMX-8 was comparable to that of methotrexate (MTX), the classic first-line anti-arthritis drug. Compared with conventional MTX and existing IL17RA-targeted monoclonal antibody therapies, WMX-8 peptide possesses unique advantages including low molecular weight and comprehensive regulatory effects on inflammation and cellular function. Despite current limitations, this work provides a novel candidate molecule for the development of peptide-based anti-inflammatory drugs and offers new insights into IL17RA pathway-targeted therapy for autoimmune diseases. After subsequent optimization and in-depth mechanistic studies, WMX-8 may become a valuable candidate molecule for developing novel anti-inflammatory agents targeting the IL17RA pathway.

## 5 Conclusion

In conclusion, the novel polypeptide WMX-8 specifically targets IL17RA and blocks the IL17A/IL17RA signaling pathway. WMX-8 significantly inhibits pro-inflammatory cytokine production *in vitro*, alleviates synovial hyperplasia, inflammatory infiltration, and joint damage in CIA rat model *in vivo*, and improves joint function, locomotor activity and general health status. Its therapeutic efficacy is comparable to that of MTX. This study demonstrates that WMX-8 is a promising peptide candidate for the treatment of rheumatoid arthritis and other IL17RA-mediated autoimmune diseases, providing a new strategy for the development of targeted peptide drugs.

## Data Availability

The original contributions presented in the study are included in the article/Supplementary Material, further inquiries can be directed to the corresponding author.
