# Supplementary material for: Anti-inflammatory effect of novel polypeptide WMX-8 through specific blockade of the IL17RA signaling pathway
Source: Front Pharmacol. 2026 Jul 24;17:1842752. doi: 10.3389/fphar.2026.1842752 (PMC13448674; doi:10.3389/fphar.2026.1842752)
Supplement: Supplementary file 1 [file Image1.pdf]

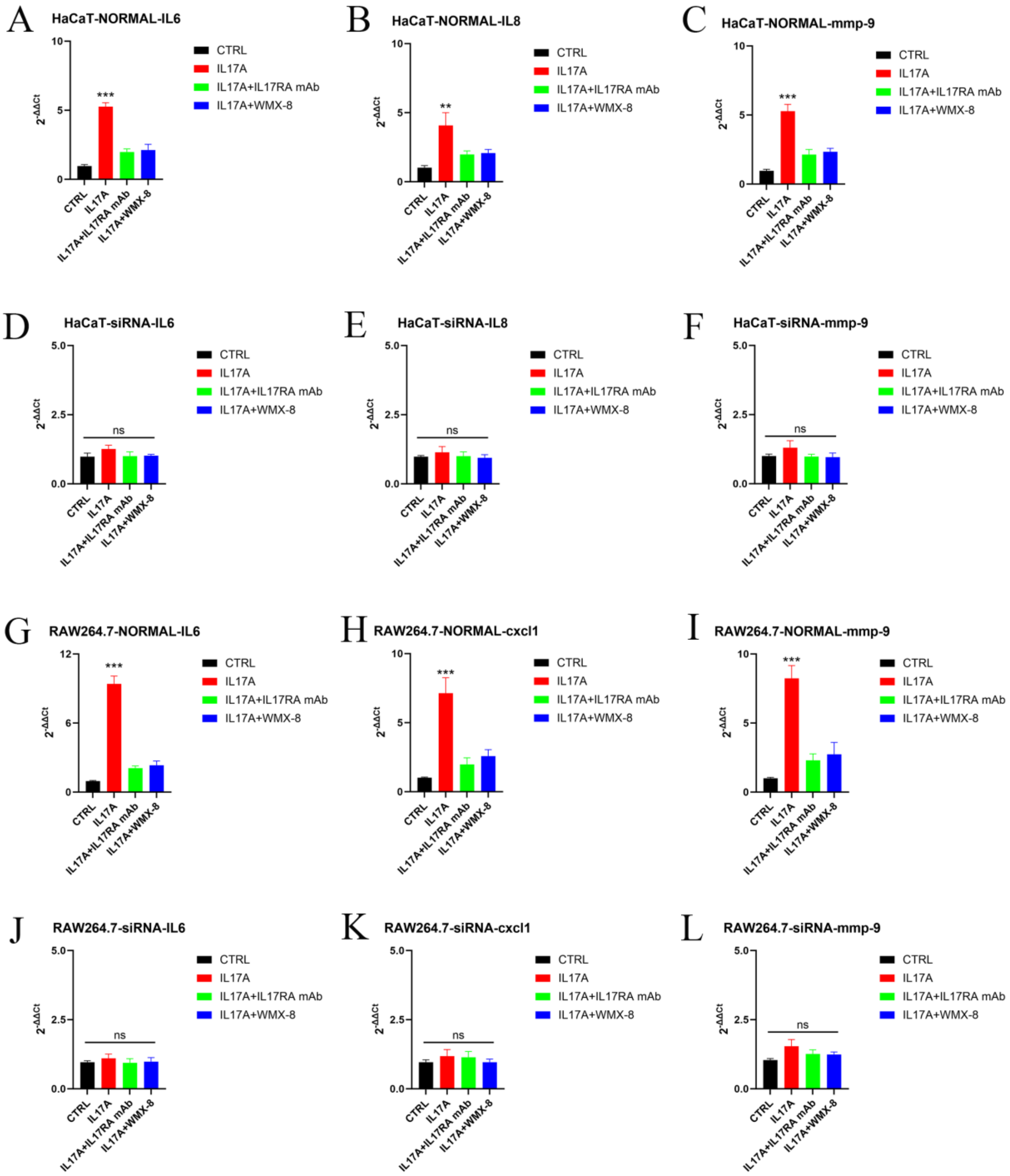


**Figure S1** QPCR analysis of relative mRNA expression of pro-inflammatory cytokines in various cells under different treatments. (A-F) Relative mRNA expression levels of pro-inflammatory cytokines in HaCaT cells (IL17RA-normal expression subtype (NORMAL) or siRNA-mediated IL17RA-deficient subtype (siRNA)) after different treatments, detected by quantitative real-time PCR (QPCR): control group (CTRL), IL17A stimulation group, IL17A combined with IL17RA monoclonal antibody group (IL17A+IL17RA mAb), and IL17A combined with WMX-8 peptide group (IL17A+WMX-8). Among these, (A-C) correspond to the NORMAL subgroup of HaCaT cells, detecting the mRNA expression of IL-6, IL-8, and MMP-9, respectively; (D-F) correspond to the IL17RA-siRNA subgroup of

HaCaT cells, with the same detection indicators as above.(G-L) Relative mRNA expression levels of pro-inflammatory cytokines in RAW264.7 cells (IL17RA-normal expression subtype (NORMAL) or siRNA-mediated IL17RA-deficient subtype (siRNA)) after the above four treatments, detected by QPCR. Among these, (G-I) correspond to the NORMAL subgroup of RAW264.7 cells, detecting the mRNA expression of IL-6, CXCL1, and MMP-9, respectively; (J-L) correspond to the IL17RA-siRNA subgroup of RAW264.7 cells, with the same detection indicators as above.The ordinate of each subplot represents the relative mRNA expression of cytokines (expressed as $2^{\Delta\Delta Ct}$ values). Data are presented as mean ± standard error (SEM); statistical significance is indicated by * ($P < 0.05$), ** ($P < 0.01$), *** ($P < 0.001$) for intergroup differences, and ns for no significant difference ($P > 0.05$).
