# Supplementary material for: Anti-inflammatory effect of novel polypeptide WMX-8 through specific blockade of the IL17RA signaling pathway
Source: Front Pharmacol. 2026 Jul 24;17:1842752. doi: 10.3389/fphar.2026.1842752 (PMC13448674; doi:10.3389/fphar.2026.1842752)
Supplement: Supplementary file 2 [file Image2.pdf]

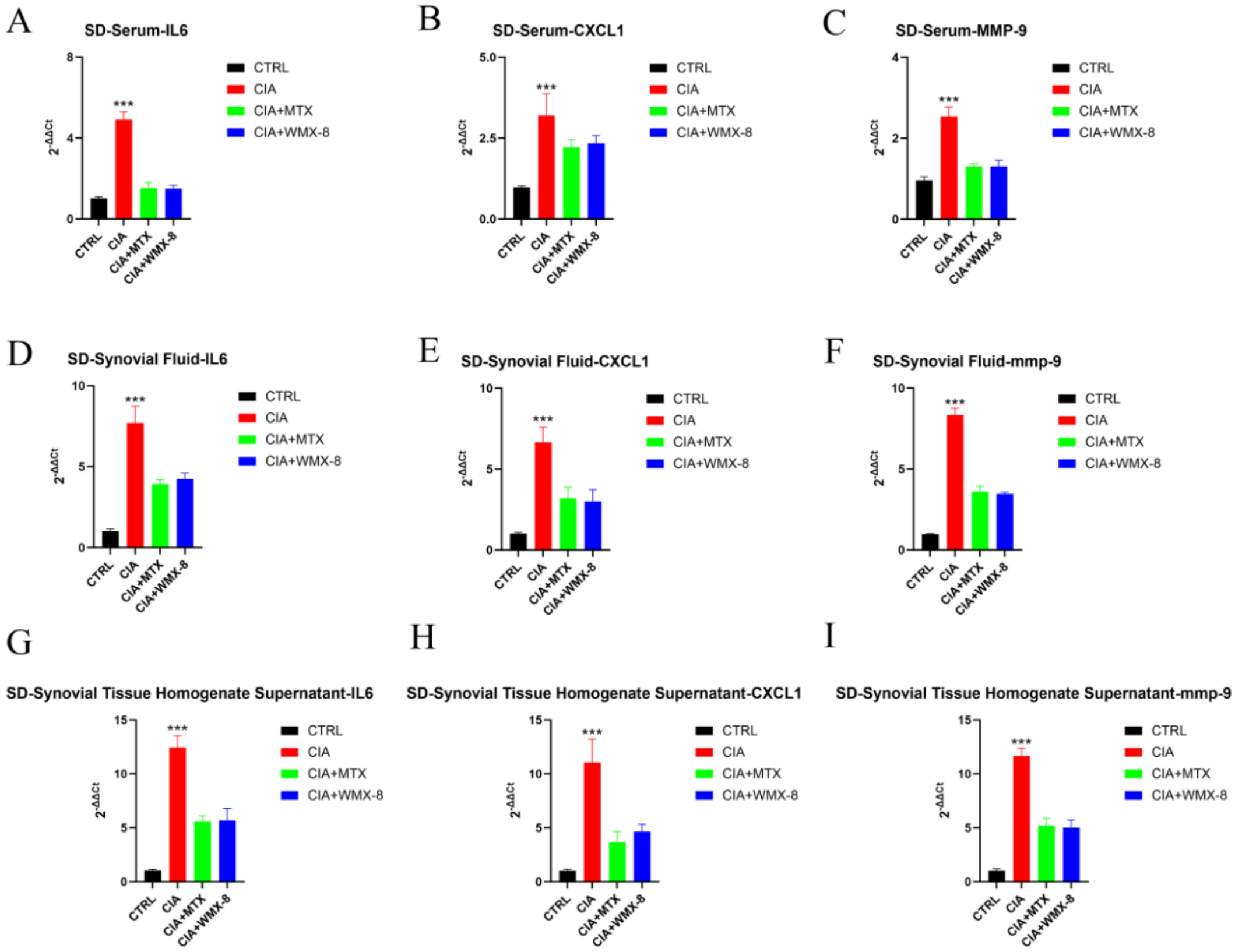

Figure S2 (A-I) Quantitative real-time polymerase chain reaction (qPCR) was performed to detect the relative mRNA expression levels of pro-inflammatory cytokines in serum, ankle joint cavity fluid, and synovial tissue of SD rats under different treatments. Among them, (A-C) show the mRNA expression levels of IL-6, CXCL1, and MMP-9 in serum samples; (D-F) show the results in ankle joint cavity fluid samples, and (G-I) show the results in synovial tissue samples (the same cytokines as those in serum). For each qPCR subfigure, the ordinate represents the relative mRNA expression level of the corresponding cytokine (expressed as $2^{(-\Delta\Delta Ct)}$ values), and the abscissa represents the days post-modeling (unit: days). Data are presented as mean ± standard error (SEM); statistical significance is indicated by * ($P < 0.05$), ** ($P < 0.01$), *** ($P < 0.001$) for intergroup differences, and ns for no significant difference ($P > 0.05$).
